# Was Aristotle right about moral decision-making? Building a new empirical model of practical wisdom

**DOI:** 10.1371/journal.pone.0317842

**Published:** 2025-01-22

**Authors:** Shane McLoughlin, Stephen Thoma, Kristján Kristjánsson

**Affiliations:** 1 Jubilee Centre for Character and Virtues, University of Birmingham, Birmingham, United Kingdom; 2 Department of Educational Psychology, University of Alabama, Tuscaloosa, Alabama, United States of America; Purdue University, UNITED STATES OF AMERICA

## Abstract

This article presents the development and validation of the Short *Phronesis* Measure (SPM), a novel tool to assess Aristotelian *phronesis* (practical wisdom). Across three studies, using large, nationally representative samples from the UK and US (demographically matched to census data), we employed a systematic and rigorous methodology to examine the structure, reliability, and validity of the SPM. In Study 1a, exploratory factor analysis identified ten distinct, internally reliable components of *phronesis*, challenging the traditional four-component Aristotelian model. Study 1b confirmed these findings in two additional nationally representative samples from the UK and the US. In Study 1c, the SPM demonstrated strong test-retest reliability over two months. Study 2 used network analysis to uncover interrelations among the components, allowing for the creation of a new and empirically driven *neo*-Aristotelian model of *phronesis*. In Study 3, we tested criterion validity, showing *phronesis* correlates positively with flourishing and predicts flourishing two months later, demonstrating strong predictive validity. *Phronesis* also correlated with Big 6 and Dark Tetrad personality traits, moral disengagement, and Moral Foundations in expected directions. Importantly, *phronesis* predicted key outcomes—related to flourishing, moral disengagement, and morally relevant aspects of personality—beyond what Moral Foundations alone explained, with an average increase in predictive power of 13.7% across all outcomes. The SPM is quick to administer (15–20 minutes), making it a valuable tool for researchers and practitioners in psychology, education, and professional ethics. The introduction of the neo-Aristotelian *Phronesis* Model, and the identification of central *phronesis* components, offers actionable insights for moral psychologists and moral educators, suggesting areas of focus that could yield broad, positive effects across related traits, providing a significant contribution to both theory and practice.

## Introduction

You find yourself at a crossroads: whether to report a close male colleague’s inappropriate remark about female co-workers, or to remain loyal to a long-standing friendship. On one hand, professional integrity and respect for others demand transparency and accountability. On the other, loyalty and personal trust suggest restraint, recognizing that people can make mistakes. In an age where every action is scrutinized, are we bound to prioritize ethical duty over personal bonds? Or does the pursuit of justice risk eroding the very relationships that hold us together? Quandaries like this represent the rough and tumble of moral life for those who are committed to trying to make the right moral choices. Moralists, authors of popular fiction, and scriptwriters of films and soap operas tend to be obsessed with the dichotomy between saints and sinners: the “goodies” and the “baddies.” However, moral philosophers and moral psychologists have historically been sensitive to the fact that most ordinary “decent” people are not wrestling with the fundamental question of whether to be good or bad: they are, however, constantly struggling with quandaries in which two or more moral virtues seem to clash. Kohlberg’s [1] whole research agenda in moral psychology was premised on the assumption that its biggest question was about how to bridge the gap between wanting the good and doing the good [2]. Much earlier, the progenitor of current virtue-based moral philosophy, Aristotle, had mostly ignored the “baddies”—as beyond redemption—and focused on offering guidance to his readers who had been “brought up in good habits” ([3], pp. 6; 292 [1095b4–5]; [1179b11–31]).

Aristotle famously posited *phronesis* (practical wisdom) as directing the whole virtue orchestra: a metacognitive intellectual virtue that oversees the moral values and virtues and provides them with the necessary checks and balances to secure overall ethically wise decisions. It is concerned both with finding the correct “golden mean” (i.e., the medial state between deficiency and excess) of a single virtue, when applied in complex contexts, and the proper interactivity, or balancing, of different virtues where those seem to call for different responses in the same situation. For instance, is it possible to reconcile somehow the commonly clashing demands of honesty and kindness, by being kindly honest or honestly kind, or may a particular situation call for the abandonment of one of the two virtues? Whatever the eventual decision may be, it is the faculty of *phronesis* that is meant to do the adjudicative heavy lifting. At the same time, it turns simple dispositions to be good along various independent paths into a coherent way of life, a comprehensive moral journey, guided by right reason, albeit a journey that is heavily contextualized and individualized [4].

*Phronesis*, or practical wisdom, has been undergoing a significant academic revival of late. What we could call the new “*phronesis* bandwagon” was originally driven by philosophers [5–8], but this interest has also percolated to psychologists [9–12] and theorists working within various branches of professional ethics, such as medicine [13], nursing [14], and business [15]: branches where *phronesis*-guided virtue ethics is gradually becoming the moral theory of choice. Various interdisciplinary studies are also emerging [4]. Saliently for the present context, a long-standing discourse on general wisdom within psychology has also been swaying significantly in the direction of *phronesis*—away from both *sophia* (theoretical wisdom) and *deinotes* (wisdom understood as mere instrumentalist calculation). Grossmann et al.’s [16] landmark paper and all the responses to it in the same issue seem to be turning the tide in psychological research on wisdom toward a sharper focus on the moral aspirations undergirding wisdom—and hence toward an alignment with the neo-Aristotelian *phronesis* tradition.

Philosophy has a reputation for being a cloistered discipline with little interest in collaboration with social science. Neo-Aristotelian virtue ethics constitutes an exception because of its naturalistic methodology, according to which all ethical theorizing must be informed by empirical evidence [7]. On the other hand, psychology has also arguably been too cloistered as a discipline in the field of wisdom research, where comprehensive overviews continue to be written [17, 18] without taking account of philosophical work on practical wisdom or, more generally, the collective historical tradition within fields such as philosophy and theology of trying to make sense of what wisdom is. Despite the recent interest within psychology [16] in an alignment between philosophical and psychological accounts of wisdom, prior to work in the present research center, no psychologically credible conceptualization of *phronesis* existed and no instrument to measure it [9, 19]. A core assumption of the current research study is that philosophers and psychologists studying wisdom are ultimately interested in more or less the same thing, and that crossover work between them can be fruitful. We return to that assumption in the General Discussion.

### Some historical and current competitors

The perennial importance of excellence in moral decision-making explains why *phronesis* attracted attention in early moral theorizing. However, this interest gradually faded in Enlightenment and post-Enlightenment discourses, with *phronesis* being brushed off as too indeterminate as a decision procedure, and indeed as part of a naïve “bag-of-virtues” conception of moral life, according to Kohlberg [20]. The decline of interest in *phronesis* developed in tandem with the erosion of virtue ethics as a paradigm in moral philosophy [21] and the replacement of “character” with a conception of human “personality” as “character devaluated” in psychology [22]. *Phronesis*, with its emphasis on making wise moral decisions based on the specifics of a context (situational and individual), cannot be formulated via the kind of algorithmic principle-based decision-making typically favored by Enlightenment and post-Enlightenment thought.

As a decision process, *phronesis* thus became replaced by top-down procedures that better fit the post-Enlightenment frame of mind. There are many historical influences cast in the mold of that thought, including an instrumentalist cost-benefit analysis of the utilitarian kind [23, 24], a formalistic deontological (rule-based) procedure emphasizing purely rational arbitration of decision-making [20, 25], a sentimentalist philosophy that views desires and emotions (not reason) as the essential sources of decision-making [26], and a logical positivist philosophy of science that eschews values and ethics in science (critiqued by Richardson et al. [27]). These influences on psychology were all unfriendly to the concept of *phronesis*. In the flow and ebb of intellectual opinion, these post-Enlightenment positions have come under heavy criticism for their uncritical bifurcation of facts and values [28–30]. At the same time, the rationalistic approach in early moral psychology [20] also suffered a setback when it transpired that correlations between developmental stages of moral reasoning and actual moral action were low [2, 31].

While neo-Kohlbergians and other psychologists have come up with complex models to show how various psychological functions might combine to aid moral decision-making [31, 32], the most heavily cited accounts of the moral life in current psychological circles are surprisingly reticent on the problem of virtue conflicts within an individual’s moral make-up. The Values-in-Action taxonomy of 24 character strengths and virtues favored by positive psychologists [33] makes do without any integrative meta-virtue. When pressed, defenders of this model argue that three of the 24 strengths listed—namely prudence, perspective, and judgment—combine to fulfill the required synthesizing role [34], but that begs the question of who or what calls the shots if these three collide. This problem is compounded by the lack of any golden-mean architectonic in the positive psychological system, assuming rather that the more of each virtue is better [35]. Moral Foundations Theory has provided valuable insights into the way in which liberals and conservatives prioritize different values and virtues, with the former foregrounding care and fairness (understood as equality), but the latter fairness (understood as proportionality), loyalty, authority, and purity [36]. However, the theory has so far provided little insight into how, say, conservatives adjudicate conflicts between proportionality and loyalty.

Some psychologists critique the *phronesis* construct as developmentally and educationally underdeveloped and propose, rather, reliance on better entrenched developmental constructs such as that of metacognition [37]. The recent buzz about *phronesis* in philosophical circles notwithstanding [38], some philosophers have also toyed with the idea that *phronesis* may be a redundant concept [38, 39], while others argue that standard Aristotelian approaches do not give *phronesis* sufficient priority with respect to the moral virtues (as traits), and that the only trait a truly virtuous moral agent needs to possess is simply *phronesis*. More specifically, on this view (the so-called Aretai Center Model) all ethical virtues are ultimately unified within *phronesis* itself, understood as overall moral *expertise* [40].

We return to some of those alternative conceptions, in light of our own findings, in the General Discussion. At the present juncture it is worth pointing out, however, that some of the most recent evidence from psychological studies casts doubt on a redundancy thesis about a synthesizing virtue such as *phronesis*. For example, Feraco et al. [41] found that the VIA character strengths aggregated into a single character factor using a bifactor model, with several specific character strengths not predicting life satisfaction and mental health over and above the general factor. The authors also emphasize the need to better understand what that general factor represents and how these character strengths are integrated to affect external outcomes. Other researchers such as Han [42] have examined the network structure among moral functioning components and concluded that functional connectivity between components may be important, as these are, for instance, tied to civic engagement levels. Finally, Han [43] examined whether neuroscientific evidence can support standard models of *phronesis*, focusing on network-based moral functioning at the neural level and its implications for *phronesis* eliminativism. He proposed that while evidence supports the standard *phronesis* models, future studies should more directly target *phronesis* as a construct, acknowledging the limitations of current neuroscientific approaches in fully capturing its multifaceted nature. Overall, there is reason to suppose that a standard model of *phronesis* may indeed be substantiated psychologically; yet this requires an integrative approach that encompasses both structural psychometric and network approaches to better comprehend its complex and multidimensional character.

#### Introducing and unpacking the Aristotelian “standard model” of *phronesis*

Given the historical role of Aristotle as the forebear of all contemporary philosophical and psychological work on (practical) wisdom, we believe his theory deserves serious scientific exploration; hence the original question in the title of this article. In this section, we explore the theory in more detail, before later subjecting it to a comprehensive empirical investigation.

In Aristotle’s [3] ethical system, *phronesis* is nothing less than the lynchpin of a flourishing life, actualizing the virtues and representing good character. According to Aristotelian character developmental theory, young people who have acquired the right moral habits through good upbringing need to gradually develop this intellectual virtue to guide their decision-making. Otherwise, their moral lives will be fragmented, uncritical, and lacking in intrinsic value. Rejecting Plato’s idea of a moral *master virtue* (justice), which trumps the other virtues in times of conflict, Aristotle proposed this intellectual *meta-virtue* instead. In that sense, then, Aristotelian *phronesis* is best understood as deliberative excellence in moral decision-making [4].

It is important to note at this point that the foundational concept of Aristotelian and neo-Aristotelian virtue theories (as well as their educational incarnations as “character education”) [7, 44] is not “character” or even “virtue,” but rather “flourishing.” Flourishing is seen as the “ungrounded grounder” of the good life [33], which the other key capacities are either conducive to or constitutive of. Although flourishing (often under the banner of “objective wellbeing”) has been on psychological agendas for decades, its prominence has been on the rise in recent times, as well as efforts to measure it [45, 46]. Although the immediate aim of *phronesis*, on an Aristotelian understanding, is excellence in moral decision-making, its ultimate aim is not just “prosocial behavior” (an end in which virtue ethicists tend to be only derivatively interested) but rather its constitutive contribution to an overall flourishing life. The overarching research question motivating the present research project, to which the question in the title refers, is whether *phronesis* predicts the core components of flourishing.

There are already large theoretical literatures on practical wisdom in general [8] and the Aristotelian conception in particular (see [6]). However, most of those literatures are either exegetical or purely philosophical in orientation, and hence outside of our immediate practical and empirical interests. What matters for present purposes is that in philosophy there has gradually evolved what Miller [39] calls a neo-Aristotelian “standard model of *phronesis*,” which carries independent interest, whatever one may think of some of Aristotle’s own claims. In the standard neo-Aristotelian model, the task of *phronesis* is complex [47], and a common suggestion from the theoretical literature is that it has at least three functions. First, *phronesis* helps us spot situations where the relevant virtue is required and how to execute it. For example, courage is the virtue that is appropriate to situations involving risk. Second, *phronesis* allows us to integrate different virtues that seem to come into conflict in the same situation, such as being courageously generous. This arbitration can also lead to enacting one virtue that is a higher priority and in unresolvable conflict with a second virtue (e.g., mercy versus justice). Only through *phronesis* do the virtues become a “package deal” ([8], p. 26). Third, the *phronimos* (person possessing *phronesis*) re-evaluates and regulates emotional traits acquired early in life, infusing them with reason and justification. Other scholars have added a fourth function, of “deep understanding” [47] of the human condition, to this mix: an understanding of what constitutes human flourishing as an irreducibly moral activity.

Although it has been suggested that Aristotle’s remarks on *phronesis* are not always particularly illuminating, especially from a contemporary developmental and educational perspective [37], it does seem possible to derive a general account of *phronesis* from those texts that emphasize its diverse above-mentioned *functions*—hence refining and concretizing “the standard model.” The best way to convey the nature of those functions in contemporary psychological language is to say that the construct is made up of various (inter-related) *components*, and we will hence shift to talking about “components” rather than “functions” in what follows. Therefore, we hypothesize, in line with previous theoretical writings [4, 9, 19], that there are *four* psychometric components of *phronesis*, reflecting the four functions they are theoretically tasked with carrying out. The four-componential version of the “standard model,” which we call the Aristotelian *Phronesis* Model (APM), constitutes a hypothesis derived from Aristotelian theory, a hypothesis tested within Study 1. Moreover, the components do not refer to psycho-moral capacities that are completely independent of one another and can be turned “up” or “down” in isolation; rather, they are inter-related as explained below (see further in [4]). The four APM components are outlined below.

#### Moral perception

*Phronesis* in the APM involves the cognitive discriminatory ability to perceive the ethically salient aspects of a situation and to appreciate these as calling for specific kinds of responses [9]. In the *phronimoi*, this becomes a moral cognitive excellence in that, after having noted a salient moral feature of a concrete situation calling for a response, they will be able to weigh different considerations and see that, say, courage is required when the risk to one’s life is not overwhelming but the object at stake is extremely valuable; or that honesty is required when one has wronged a friend. Herein, we adopted *Moral Perception* as a more generally understood term. We could also refer to this component as *moral sensitivity*, a term often found within standard moral psychology/education literatures [31].

#### Moral emotion

Individuals foster their emotional wellbeing through *phronesis* by coordinating their emotional responses with their understandings of the ethically salient aspects of their situation, their judgment, and their recognition of what is at stake [9]. This is partly because they will have developed habituated virtues, meaning, *inter alia*, that their emotions have been shaped to align with the motivations and behaviors characteristic of a virtuous person. Additionally, these emotional habits are reinforced and solidified through understanding and reasoning, providing a robust intellectual foundation for their responses. For example, a *phronimos* might recognize that her appraisal of the situation is problematic, giving rise to an emotional response that is inappropriate to the situation. The emotion-regulative component can then help her adjust her emotion by, for instance, giving herself an inner “talking to” or asking herself questions about what is prompting the ill-fitting emotional response. For this reason, we can also refer to this component, in a more standard Aristotelian way, as infusing emotion with reason [48]. The term we will use hereinafter is *Moral Emotion*, encapsulating the emotional regulative function emphasized by philosophers, and the positive and negative emotions associated with moral actions.

#### Moral identity

The synthesizing work of *phronesis* operates in conjunction with the agent’s overall understanding of the kinds of things that matter for a flourishing life: a person’s own ethical aims and aspirations, her understanding of what it takes to live and act well, and her need to live up to the standards that shape and are shaped by her understanding and experience of what matters. This amounts to what we call a blueprint of flourishing [9]. A “blueprint” has more similarity to what psychologists call “moral identity” than a full-blown theoretical outline of the good life [49, 50], and we use *Moral Identity* for simplicity in subsequent sections. *Phronetic* persons possess a general justifiable conception of the good life (*eudaimonia*) and adjust their overall reactions to that blueprint, thus furnishing it with motivational force. This does not mean that each ordinary person needs to have the same sophisticated comprehension of the “grand end” of human life as a philosopher might have in order to count as possessing *phronesis*. Rather than being an “elite sport,” the sort of grasp of a blueprint of the aims of human life informing *phronesis* is within the grasp of the ordinary individual. It draws upon the person’s standpoint on life as a whole and determines the place that different goods occupy in the larger context.

#### Moral adjudication

Assume that we have identified a moral problem correctly as one potentially requiring input from two or more apparently conflicting moral virtues. Let us further assume that we have infused our relevant emotions with reason and that they are not obstructing the decision process. Finally, assume that we have a clear, non-self-deceptive identity of who we want to be—a blueprint of the good life—and an overall motivation to bring our reactions into line with that identity. That leaves just the final component of the four-componential construct: the integrative component—what we could also call its adjudicative component [9] or, in line with standard moral psychology, simply denote as a form of “moral reasoning.” Through this component, an individual integrates different virtue-relevant considerations, via a process of checks and balances, especially in circumstances where different ethically salient considerations, or different kinds of virtues or values, appear to be in conflict and agents need to negotiate dilemmatic space. For the sake of simplicity, we will use the term *Moral Adjudication*, hereinafter.

The APM is built upon the foundational work of the *Jubilee Centre Phronesis Report* [51] and the influential framework of Darnell et al. [9, 19]. These earlier studies provided a pivotal theoretical base and important preliminary empirical insights into the structure of *phronesis*. A particular strength of Darnell et al.’s [19] work was its use of a confirmatory factor analysis (CFA) within a structural equation modeling framework to assess the viability of the four-component APM. By drawing on existing validated measures aligned with the theorized components of *phronesis*, this study offered an innovative first attempt to operationalize the APM and demonstrated that a second-order model, with four interrelated components feeding into an overarching *phronesis* construct, could be empirically supported.

However, while this approach provided strong theoretical and empirical groundwork, it also presented certain limitations that future research needed to address. First, by relying on existing measures originally developed for other purposes, the study risked a degree of conceptual misalignment between the measures and the specific nuances of the APM components. Second, the use of CFA, while appropriate for testing a pre-theorized model, meant that the data had less opportunity to inform the factor structure inductively. Third, the resulting measure took approximately 45 minutes to complete (hereinafter referred to as the Long *Phronesis* Measure, or LPM), raising concerns about its practical feasibility for large-scale studies or applied settings. Fourth, the participants were not representative of the general population, so the data could not speak to *phronesis* in general. Finally, the item set and scoring instructions were not made fully accessible, which limited the replicability of the findings and constrained the development of a broader empirical program of research on *phronesis*.

These strengths and limitations highlight both the significance of the Darnell et al. [19] study and the need for further work to build upon its contributions. Future research required measures that were specifically tailored to reflect the APM framework, an approach that would balance theoretical precision with practical utility. Additionally, an inductive exploration of the dimensions of *phronesis*—through methods such as exploratory factor analysis (EFA)—would allow the data itself to reveal the structure of the construct, providing a stronger empirical foundation for subsequent confirmatory analyses. Addressing these issues is essential to advance both the theoretical understanding of *phronesis* and its measurement in a way that supports scalable, replicable, and substantive empirical research.

### The current study

Our study traverses two culturally distinct countries—the United States and the United Kingdom—by drawing on representative adult samples. This breadth of inquiry allows us to move beyond philosophical conceptions of *phronesis* towards an empirical psychological account representative of these general populations. We are also interested in the extent to which this inquiry enables us to respond to the sort of eliminativism about *phronesis* mentioned earlier [37, 39] as well as to alternative non-Aristotelian conceptions [40]. We return to those questions in the General Discussion.

In addition to examining the internal structure of *phronesis*, our research delves into its intersections with core psychological constructs, including personality traits and so-called Moral Foundations. This aspect of the study enriches our understanding of how *phronesis* interacts with fundamental human attributes and moral reasoning processes. The research questions driving this work are broadly designed to develop and assess a new measure of the APM, with the subsequent goal of understanding the workings and predictive power of Aristotelian *phronesis* as such. This overall focus can be subdivided into a measurement development phase, in which we test whether the four theorized functions of *phronesis* within the APM emerge from a bottom-up analysis, followed by studies assessing the validity of the measure in relation to a wide range of psychologically and sociologically significant variables. Study 1 addresses the measurement properties of the new instrument using a mix of exploratory (Study 1a) and confirmatory approaches (Study 1b) in a sample collected in the UK and in the USA. While it is also important to research practical wisdom in non-WEIRD contexts [52], establishing a general understanding of *phronesis* within Western culture, from which the concept originally emerged, was seen as an appropriate first step for a substantive research program. This phase of the paper concludes with Study 1c where information is presented about the test-retest reliability of the instrument. In Study 2, we explore *phronesis* and its relationship to flourishing from a network psychometrics perspective, in an attempt to determine which aspects of a network of *phronesis* sub-factors are most central. This was a necessary study to help understand how the *phronesis* components identified in Study 1 interrelate. Finally, we explore the association between *phronesis* network components and a wide range of variables of psychological and sociological interest (Study 3). In particular, we wanted to understand whether *phronesis* would predict variables of interest (flourishing, morally salient aspects of personality, and moral engagement) over and above Moral Foundations, thus testing whether this construct adds explanatory power over and above one of psychology’s premier moral theories. Taken together, the studies described in this article were designed to create an ambitious, wide-ranging, and well-validated measure of *phronesis* that would satisfy requirements made by psychologists, practically minded philosophers, and educators. More broadly, we attempted to shed a greater degree of empirical light on an important and ancient philosophical concept than has previously been achieved.

All analyses and codes are available at https://tinyurl.com/spmdevelopment for researchers to check our work for studies within this paper. This data may also be reused with the authors’ prior permission. As the measure of *phronesis* developed for this study was initially based on exploratory analyses, analyses reported herein were not pre-registered.

## Study 1: Establishing the dimensions of *phronesis*, and developing the short *phronesis* measure

### Study 1 Purpose and aims

Aristotle’s *phronesis*, or practical wisdom, has stood as a guiding concept for understanding moral reasoning and human flourishing for over 2,300 years. While rich in theoretical depth, philosophical constructs like *phronesis* often rest on untested assumptions about human behaviour. Study 1a–c sought to bridge this gap by developing the Short *Phronesis* Measure (SPM), using a systematic process to derive and subsequently test the structure of *phronesis* empirically. These studies are presented sequentially below, reflecting the order in which they were conducted, with the findings of each study building on the previous.

**Study 1a** was about impartially deriving the structure of *Phronesis* from the data. Guided by Aristotelian theory, the item development process ensured that the SPM was tethered to the underlying theory. However, EFA took an impartial look at the data to inductively uncover latent dimensions of *phronesis*, rather than imposing these dimensions in a top-down manner based on theory alone. This approach allowed for the possibility that the data might reveal nuances or even divergences from the Aristotelian framework, ensuring that the resulting measure would reconcile this long-standing theory with empirical reality, availing of modern social science methods.
**Study 1b** used CFA to evaluate whether the inductively derived structure identified in Study 1a holds up across independent UK and US samples. This phase provides a critical deductive check on the validity of the model, assessing its robustness under stricter conditions. Additionally, measurement invariance testing examined whether the dimensions of *phronesis* were consistent across cultural contexts, ensuring the measure’s generalisability.
**Study 1c** evaluated the temporal stability of *phronesis*. By conducting test-retest reliability analyses, this phase addressed whether *phronesis* behaves as a stable aspect of one’s character as Aristotle might have predicted, or whether it fluctuates as a transient state. This insight is critical for determining the SPM’s utility in intervention studies and longitudinal research. Temporal stability ensures that the measure can reliably capture enduring characteristics of practical wisdom over time.

Together, these studies provide a rigorous framework for operationalizing *phronesis*. Study 1a derives a theoretically informed yet empirically grounded structure, Study 1b tests the robustness and universality of this structure, and Study 1c ensures the measure’s stability over time. This stepwise process integrates philosophical insight with empirical testing, transforming *phronesis* from a conceptual ideal into a measurable construct that can inform research and practice.

### Study 1 Method

#### Participants

Participants for Studies 1a-1c were recruited online using Prolific Academic. Studies have shown that Prolific provides high quality data with high levels of participant comprehension and attention, and low levels of dishonesty [53]. Participants in Studies 1a and 1b were compensated for their time at a rate of £9/hr for one hour of their time, while Study 1c participants were compensated at the same rate for 20 minutes of their time. All data for this project (including subsequent studies) were collected between August 2023 and February 2024.

For Study 1a, after excluding those who elected to withdraw, did not have a valid Prolific ID, or had less than 95% survey completion, the final sample comprised 1998 participants. The sample was representative based on age, sex, and ethnicity, reflecting UK census data (see Table 1). For Study 1b, we recruited two new representative samples, one from the UK (see Table 2 for demographics) and a second from the US (see Table 3). The UK sample included 1,000 participants from Prolific Academic, with 997 retained after removing participants who wished to withdraw. Likewise, we recruited 1000 participants from the US, with 988 retained after removing participants who wished to withdraw. For Study 1c, we recruited 300 participants. Participants were only eligible to take part if they were already part of Study 1b’s UK-representative sample. We gained Study 1c participants’ permission to link their Study 1b data with their Study 1c responses, after which point their responses would be fully anonymized. After removing participants with incomplete data, the final number of participants for Study 1c was 295. The mean age of participants recruited was 45.73 (*SD* = 13.55), with 137 females, 156 males, and two identifying as “other” or “prefer not to say”. For ethnicity, 254 participants identified as “White”, 14 as “Black”, 11 as “Central Asian”, eight as “East Asian”, seven as “Mixed/Multiple ethnic groups”, and one as “Prefer not to say”.

**Table 1:** Demographics of the exploratory UK sample compared to demographic data from the Office for National Statistics (ONS) to evidence representativeness.

ONS Categories	% England and Wales	% Current Sample
Sex^1^		
Female	51%	1024 (51.25%)
Male	48%	964 (48.24%)
Other	N/A	10 (0.50%)
Age^1^		
Under 15	17.4%	N/A
15–64	64.1%	1750 (87.59%)
65+	18.6%	248 (12.41%)
90+	0.9%	0 (0%)
Ethnicity^2^		
White / White British	84.8%	1731 (86.64%)
Asian / Asian British	8%	142 (7.10%)
Black, African, Caribbean / Black Other	3.5%	60 (3.00%)
Mixed / Multiple ethnic groups	1.8%	23 (1.15%)
	1.9%	42 (2.10%)

*Note*: ONS data (2019–2021) are for England and Wales; we did not collect data on country within the UK to compare Wales, Northern Ireland, and Scotland data. Where totals do not add up to 100%, this reflects missing data; participants were allowed to skip any questions they did not want to answer. Estimates are those most readily accessible to us via the ONS pages.

^1^2019 ONS Figures: Population and household estimates, England and Wales - Office for National Statistics (ons.gov.uk)

^2^2019 ONS Figures: Population estimates by ethnic group and religion, England and Wales - Office for National Statistics (ons.gov.uk)

^3^2022 ONS Figures: Employment in the UK - Office for National Statistics (ons.gov.uk)

^4^2021 ONS Figures: Full time and part time employment - GOV.UK Ethnicity facts and figures (ethnicity-facts-figures.service.gov.uk)

**Table 2:** Demographics of the confirmatory UK sample compared to demographic data from the Office for National Statistics (ONS) to evidence representativeness.

ONS Categories	% England and Wales	% Current Sample
Sex^1^		
Female	51%	509 (51.10%)
Male	48%	482 (48.39%)
Other	N/A	5 (0.50%)
Age^1^		
Under 15	17.4%	N/A
15–64	64.1%	857 (85.96%)
65+	18.6%	137 (13.74%)
90+	0.9%	0 (0%)
Ethnicity^2^		
White / White British	84.8%	875 (87.85%)
Asian / Asian British	8%	63 (6.32%)
Black, African, Caribbean / Black Other	3.5%	30 (3.01%)
Mixed / Multiple ethnic groups	1.8%	9 (0.90%)
	1.9%	19 (1.91%)

*Note*: ONS data (2019–2021) are for England and Wales; we did not collect data on country within the UK to compare Wales, Northern Ireland, and Scotland data. Where totals do not add up to 100%, this reflects missing data; participants were allowed to skip any questions they did not want to answer. Estimates are those most readily accessible to us via the ONS pages.

^1^2019 ONS Figures: Population and household estimates, England and Wales - Office for National Statistics (ons.gov.uk)

^2^2019 ONS Figures: Population estimates by ethnic group and religion, England and Wales - Office for National Statistics (ons.gov.uk)

^3^2022 ONS Figures: Employment in the UK - Office for National Statistics (ons.gov.uk)

^4^2021 ONS Figures: Full time and part time employment - GOV.UK Ethnicity facts and figures (ethnicity-facts-figures.service.gov.uk)

**Table 3:** Demographics of the confirmatory US sample compared to demographic data from the US Census Bureau’s American Community Survey 2022 to evidence representativeness.

US Census Bureau Categories	% US	% Current Sample
Sex		
Female	50.42%	501 (50.71%)
Male	49.58%	471 (47.67%)
Other	N/A	16 (1.62%)
Age		
15–19 years	7.95%	12 (1.22%)
20–24	8.19%	95 (9.64%)
25–34	16.51%	191 (19.39%)
35–44	16.08%	174 (17.66%)
45–54	14.78%	167 (16.95%)
55–59	7.50%	114 (11.57%)
60–64	7.88%	98 (9.95%)
65–74	12.37%	115 (11.68%)
75–84	6.48%	14 (1.42%)
85+	2.25%	5 (0.51%)
Ethnicity		
White	60.88%	762 (77.13%)
Asian	5.91%	53 (5.36%)
Black or African American	12.18%	130 (13.16%)
Two or more races	12.54%	31 (3.14%)
Other	8.49	12 (1.21%)

*Note*: The population level data are taken from the American Community Survey data (2022) published by the US Census Bureau. That data is available here: https://data.census.gov/table/ACSDP1Y2022.DP05

#### Measures and item development

For the SPM, we designed a questionnaire comprising 189 items that aimed to capture various theorized components of practical wisdom. These components were identified based on an extensive review of the literature, some of which has been summarized above. Following this, the first author (a psychologist experienced in psychometric measure development) manually generated an array of possible item formats and example items. These examples, along with the definitions of the constructs they purported to measure, were entered into a Large Language Model (LLM), Open AI’s Chat GPT 4.0, where the first author generated a wide array of items of the same types. The first author suggested refinements to item exemplars where he deemed necessary (e.g., if the item array did not appear to be diverse enough to capture how someone *generally* behaves, across contexts). Once this process was complete, the item list was shared with the rest of the research team (the second author is an Emeritus Professor of Moral Psychology with expertise in measurement, and the third author is a Professor of Philosophy and originally identified the four theorized components of *phronesis* we proposed to study) for critique and feedback. If items were identified as problematic, the first author manually created refined versions of these items before generating new item variations. This process was repeated until consensus was reached that we had a list of items that (i) reflected the theorized components of *phronesis* under study, (ii) were diverse in that they could, in principle, capture how someone generally behaves, and (iii) sufficiently succinct to allow for rapid test administration. The initial questionnaire was designed to be administered online and took approximately 30–40 minutes to complete.

In the item development process, we recognized that some aspects of *phronesis* could be objectively tested. For example, the ability to discern the moral relevance of a given scenario can have a defined “correct” answer. On the other hand, certain facets of the construct are inherently subjective and best captured through self-report measures. An example of this would be the experience of moral emotions, which is intrinsically subjective and thus most appropriately assessed through self-reporting. A detailed overview of these items is provided in the subsequent sections. Before proceeding further, however, it must be acknowledged that creating items that home in on all the four components (and possible sub-components) of a *phronesis* construct is a herculean task, not least because of the lack of precedents, apart from our own previous work [9, 18]. The overarching question is always, first, whether we have identified enough sub-functions to be sufficiently reflective of each broader function of the relevant component; and second, whether we have created enough distinct observable variables (survey items) to be sufficiently reflective of those sub-functions. Working on the assumption that “perfect is the enemy of the good,” we embarked on this task seeking to strike a balance between achieving wide construct coverage and having a measure that could be completed relatively quickly and easily.

*Moral perception*. The ability to accurately perceive morally relevant elements within various scenarios is not a matter of subjective interpretation. Therefore, we incorporated two distinct types of objective assessments to evaluate moral perception. The first test focused on the recognition of whether a situation itself holds any moral relevance. The second test aims to identify the specific virtues that are at stake within those morally relevant situations. These objective tests serve as an alternative to self-report measures for this particular aspect of the broader *phronesis* construct. The items included under this Moral Perception category were chosen based on the same principles as those in the earlier *Phronesis* Project [19, 51]. However, the items in this study were designed to be more straightforward to score, as they did not include any open-ended questions. It should be noticed here that the common core in both tests is an assessment of how well the individual homes in on the moral dimension within a complex social situation. While this is not the same as identifying what would be relevant to a fully *phronetic* person, in order to develop the sensitivity of a *phronimos*, the moral learner needs to be ablein the first place—to spot and categorize situations of potential moral and characterological relevance. So, the importance here lies in what the respondents identify in the situation, not their assessment of what a *phronimos* would identify; hence the use of the first-person rather than the third-person within these item sets. Drawing on ideas from Bebeau and colleagues [55], our items focus on identifying and labelling “the moral” and being able to discount other issues. Our items economically attend to the central features of previous moral perception measures, and within our model the component they illuminate behaves much like other treatments of the construct [91].

We initially formulated 20 items aimed at assessing whether participants could discern if a decision in a given scenario would have implications for their character. Participants were guided by the following instruction: “In the upcoming section, you will encounter various scenarios, each requiring a decision. Your task is to identify which scenarios involve decisions that could influence your character. Please ponder the moral or ethical ramifications these situations could have on the individual involved.” Subsequently, participants were presented with scenarios such as: “You have found out a colleague is claiming your work as their own, but confronting them could create team tension” (character-impacting based on a virtue ethical framework) or “You are an avid reader and must choose the next book to read from a stack of equally enticing options” (not character-impacting based on a virtue ethical framework). Participants had two response options: “What I decide to do in this scenario does not affect my character” and “What I decide to do in this scenario affects my character” (note that “affects” was deliberately chosen over “demonstrates”, as in theory, who we are is an aggregate of what we do, in the first instance). Responses were scored as either correct or incorrect.

The subsequent set of items for assessing moral perception consisted of 15 moral dilemmas. In these dilemmas, participants were tasked with correctly identifying the virtues implicated in each scenario. The instruction provided was: “In this section, you will encounter various scenarios along with four character traits that may or may not be pertinent to the situation. Your responsibility is to select the two traits you believe are most relevant.” For instance, participants were presented with a scenario like: “You discover a wallet on the ground containing a substantial amount of money and the owner’s identification. You must decide what action to take. Which of the following traits are most relevant to your decision? (Select two answers from the following options).” Listed below the scenario were two virtues relevant from a virtue ethical standpoint (e.g., honesty and practicality) and two less relevant virtues (e.g., humor and resilience). Participants could select two answers, resulting in scores ranging from 0–2 for each question. We relied on a broad set of virtues (*qua* positive traits of character) here that could be deemed characterologically relevant, rather than a more narrowly construed set of standard moral virtues.

Regarding the second set of questions, it could be argued that focusing on situations eliciting more than one virtue (1) illicitly limits moral perception to the identification of dilemma-like situations, eschewing single-virtue-eliciting situations, and (2) conflates moral perception with moral adjudication (see below). However, (1) the first set of items homes in on characterologically relevant situations involving (potentially) a single virtue, and (2) the task here is not to adjudicate between or integrate virtues but simply to identify which virtues are at stake in the situation [54].

*Moral identity*. The issue of whether a participant possesses a conceptual framework for their ideal moral self is best evaluated subjectively. Consequently, for this component, we employed a conventional self-report survey methodology. Participants were presented with statements pertinent to their moral identity. In this case, there was no shortage of previous measures of moral identity, although those have not been related specifically to *phronesis*, and we drew upon many of those for enlightenment [55]. The items generated aimed to emphasize the relationship between moral identity and moral decisions more concretely than found in other popular measures. For example, one of our items was “When faced with challenging situations I ask myself what a good person would do”. In contrast, popular measures like the Moral Identity Scale [56] include items that are less focused on decision making, such as “It would make me feel good to be a person who has these characteristics”. To be sure, the fact that a decision will (or will not) affect a person’s character is only one way that something could be morally relevant. An alternative approach would be to provide a scenario in which respondents must decide what to do both before and after some event (either morally relevant or not relevant) is introduced. However, as we are interested in moral identity and moral decision-making on a virtue ethical understanding—but not for example a utilitarian one of good moral identity simply as a generator of “prosocial” responses—we opted for the former understanding. Responses were collected using a five-point Likert scale, ranging from “Strongly Disagree” to “Strongly Agree.” Given that we used a single item format for this hypothesized component of *phronesis*, we initially generated an extensive set of items, totaling 25. These items were modeled after established moral identity questionnaires, maintaining consistency with the format used in previous research, including the earlier *Phronesis* Project.

*Moral emotion*. Moral emotion is also subjective, making self-report measures a suitable method for capturing this component. Additionally, moral emotion is theoretically multi-faceted, encompassing the emotions one experiences when acting morally or immorally, as well as one’s ability to regulate these emotions. Therefore, we incorporated multiple item formats to adequately assess this aspect of *phronesis*. In this way, we improved upon previous measures of moral emotion in the context of *phronesis* measurement, which focused more so on empathy and perspective taking [57].

Initially, we developed items to gauge the emotional responses individuals might experience when acting either morally or immorally in various scenarios. Participants received the following instruction: “Below are different scenarios where you decide to take specific actions. If you were to take these actions, how would you feel about yourself?” This was followed by a set of 20 statements, half of which depicted morally upright actions (e.g., “A stranger drops a £100 note without noticing. You pick it up and return it to them”), and the other half illustrated moral failings (e.g., “You exaggerate an issue at work to harm a colleague’s professional reputation”). Participants indicated their emotional response on a five-point Likert scale, ranging from “Extremely Bad” to “Extremely Good.”

While emotional regulation has both subjective (e.g., personal emotional experience) and objective (e.g., observable externalizing behavior) elements, we opted for self-report measures. This decision was made because it would be neither practical nor ethical to objectively assess emotional regulation in stress-inducing situations. Participants were guided by the following instruction: “Below, you will encounter various scenarios that may elicit an emotional response. For each situation, please reflect on how you would typically react and rate your ability to manage your emotions. By ‘manage,’ we mean your capacity to prevent your emotions from overwhelming you and to maintain your composure.” Participants then responded to 20 scenarios, some involving moral transgressions against them (e.g., “A stranger is rude to you in a public place for no apparent reason”) and others involving everyday frustrations (e.g., “You accidentally spill a drink on your clothes just as you are about to leave the house”). The emotional responses that might be regulated in these scenarios ranged from jealousy/envy (e.g., scenarios involving recognition given to someone else for your achievement.), irritation/annoyance (e.g., scenarios involving minor inconveniences, such as someone pushing in a queue or a neighbor playing loud music), anxiety (e.g., waiting for a bus that’s late or facing a cancelled flight), hurt/resentment (e.g., harsh criticism for a small mistake or a co-worker making a joke at your expense). In these scenarios, we avoided specifying a “correct” emotional response, as we were not concerned here with adjudicative capacities, and instead aimed to draw on participants’ self-knowledge about their ability not to make an obviously “incorrect” decision based on emotional impulse. Responses were collected on a five-point Likert scale, ranging from “Very Poor” to “Very Good.” We contend that the items selected under “Emotional Regulation” offer a more nuanced reflection of the construct in question compared to the generic measures of empathy used in the previous *Phronesis* Project [19, 51]. Specifically, these items target the regulatory aspect of *phronesis* more directly, rather than focusing solely on the general capacity to experience emotions.

*Moral adjudication*. Moral adjudication entails the integrative process of arriving at a “correct” moral decision through thoughtful deliberation. This concept comprises two elements: firstly, the selection of the correct moral choice based on some criterion, and secondly, the methodology employed in making that choice.

For assessing “correct” moral choices, participants were instructed as follows: “In the upcoming section, you will encounter a series of scenarios. Each scenario presents two primary considerations that represent different potential responses. Your task is to decide how you would balance these considerations if you were to act in these scenarios. You will have seven boxes to choose from. The first and seventh boxes contain the two main considerations. Selecting either of these boxes indicates that you would focus solely on that consideration, disregarding the other. The intermediate boxes signify varying degrees of balance between the two considerations. Although real-life situations may involve more than two considerations, for the purpose of this exercise, please make your decision based on the two presented.” Participants then responded to 22 items, each accompanied by a 7-point scale. Scoring was conducted in three distinct ways: The first scoring method considered “4” as the “correct” answer, representing a balanced consideration of self and others, in alignment with virtue ethical assumptions. The second method compared the average flourishing scores for each scale point. The point with the highest average was deemed “correct,” and participants were scored based on their distance from this point. This approach allowed for situational variability and was grounded in Aristotelian virtue ethics. The third method simply scored answers on a 1–7 scale, with higher scores representing greater prosociality.

The most practical way to assess moral deliberation was to ask participants to self-report on how they gather and verify information relevant to both general and moral decision-making (e.g., “I evaluate the reliability and credibility of the information sources before making a judgment”). They responded to 23 statements on a five-point Likert scale, ranging from “Strongly Disagree” to “Strongly Agree.”

To assess the extent to which people make an effort to integrate different *phronesis* components in decision-making, participants were presented with 17 statements and asked to agree or disagree on a five-point Likert scale (Strongly disagree–Strongly agree). For example, the statement “In making a decision, I consider my thoughts, feelings, and the situation at hand” gauges the application of moral emotions in a context-sensitive manner.

The final way we attempted to measure moral adjudication was with a dilemma taken from the Adolescent Intermediate Concept Measure (AD-ICM). The AD-ICM assesses the moral thought processes of young people, in particular, focusing on their transition from self-centered to conventional thinking from a neo-Kohlbergian perspective [58]. Participants use a 5-point scale to rate the morality of potential actions and underlying reasons for those actions that a main character in a story in which a moral dilemma is presented could make. Scoring is based on how closely participants’ choices align with expert judgments. Although the AD-ICM was included as it was part of our previous Long *Phronesis* Measure (LPM, see [19]; note that the LPM included two moral dilemmas, rather than one), it was simplified for scoring purposes through a Microsoft Excel file with embedded macros developed by the second author, in line with our aim to produce a practical and easy-to-score measure.

*Flourishing*. The Wellbeing Assessment (WBA; [59]) is an instrument designed to gauge comprehensive wellbeing. It is rooted in a theoretical framework that views human flourishing as a state where all aspects of life are positive. This conceptualization aligns with the World Health Organization’s holistic definition of health, which includes mental, physical, and overall wellbeing, and is also reasonably close to a neo-Aristotelian conception of flourishing [51]. The WBA assesses six domains: emotional health, physical health, meaning and purpose, character strengths, social bonds, and financial stability. Although self-reported, the WBA’s sub-scales have demonstrated predictive validity for more objective criteria. For example, the “physical health” self-report subscale has been shown to predict medical diagnoses and insurance claim data during the validation study [59]. In accordance with neo-Aristotelian virtue theory, we deemed it essential to link the adjudicative function of *phronesis* directly to flourishing and to explore participants’ deliberative strategies. The items selected under “Moral Adjudication” were more diverse than those in the earlier *Phronesis* Project, which solely used the AD-ICM.

#### Procedure

Data collection for Study 1a involved all 189 items generated for the initial practical wisdom measure’s dimension reduction phase. For Studies 1b and 1c, participants completed only the abbreviated and final 107-item practical wisdom measure following dimension reduction in Study 1a. All participants in all studies completed the WBA. Study 1b participants completed the additional measures for purposes of establishing criterion validity that were not used; this is detailed in Study 3.

Data collection was entirely conducted online. Participants filled out the relevant array of questionnaires for each study within a single session, lasting up to one hour for Studies 1a (large practical wisdom item array and the WBA) and 1b (final practical wisdom measure, the WBA, and criterion validity measures), and 20 minutes for Study 1c (reduced practical wisdom measure and the WBA only). The sequence of the questionnaires was consistent for all participants. However, within each questionnaire, the questions were presented in a randomized order, with the exception of the AD-ICM items in Study 1a, which necessitated sequential completion. Prior to beginning the questionnaires for each study, informed consent was secured from all participants.

#### Ethics

This study and other studies hereinafter received ethical approval by the University of Birmingham’s institutional review board in June 2023 (ERN_1043-Jun2023), with a subsequent amendment approved in December 2023 (ERN_1043-Dec2023). Informed consent was sought for all participants for this and subsequent studies: Participants were presented with an information page, and electronically signed a consent page if they wished to proceed with the study. No participant deception was involved. All surveys were completed online, with participants opting in of their own volition after seeing the study advertisement.

#### Analytical strategy

*Study 1a*. We initiated our analysis with EFA to empirically determine the number of factors represented by the 189 items, without imposing our preconceived theoretical structure (APM) on the data. To do this, we employed the *psych* [60] and *gparotation* [61] packages within R Studio [62]. Carpenter’s [63] recommendation for the participant:item ratio is 10:1, which we exceeded. The Kaiser-Meyer-Olkin (KMO) measure stood at .94, confirming adequate sampling for the EFA. Additionally, Bartlett’s test was conducted, and a clause was incorporated into the code to halt the EFA if the test value was less than or equal to .05. Depending on the data’s skewness and kurtosis, we employed either Principal Axis Factoring (if skewness was > 2 or kurtosis was > 7) or Maximum Likelihood as the extraction method. The number of factors to retain was ascertained through parallel analysis. Finally, the issue of whether the factors should be correlated was not immediately evident to us. This uncertainty was particularly relevant given the likelihood of a multi-factor solution, a scenario amplified by the large sample size and extensive item pool. To address this, we employed the Promax rotation method. Promax initiates with an orthogonal Varimax rotation, followed by an axis “tilting” to permit obliqueness, rather than pursuing an oblique solution directly as an Oblimin rotation would do. The computational simplicity and speed of Promax make it particularly advantageous when managing a large number of factors. Loadings below .4 for each of these factors were suppressed.

*Study 1b*. Items that were kept from Study 1a underwent a CFA using the *lavaan* [64] R package to test the anticipated factor structure within a different yet analogous sample. We used a Diagonally-Weighted Least Squares estimator as our items included non-normal ordinal responses, and robust standard errors to provide a more accurate estimation under these conditions. Consistent with the previous analysis, this CFA was adequately powered, maintaining more than ten participants for each item in the model. Aside from this, we conducted measurement invariance tests in R to understand whether configural, metric, and scalar invariance would be found across the UK vs US data.

*Study 1c*. We sought to establish test-retest reliability by correlating their scores on the SPM at the time of Study 1c with their scores approximately two months later. This would help us to understand whether scores were stable over time.

### Study 1 Results

#### Study 1a: Dimension reduction using EFA

**Fig 1:**
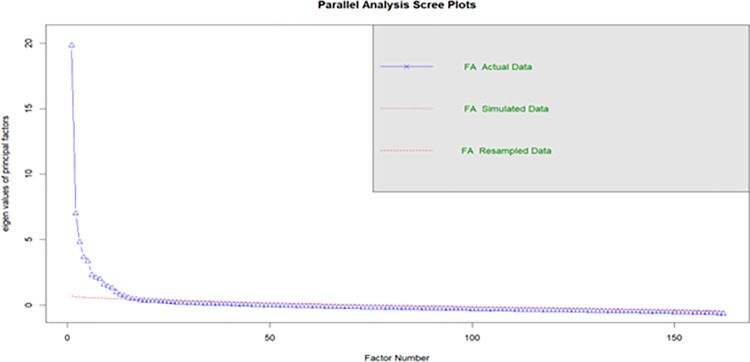
Scree plot for the parallel analysis.

Overall, parallel analysis led to the extraction of 17 factors (*RMSEA* = .02, *TLI* = .90; *χ^2^*[10716] = 107.16, *p* < .001). Among these, the initial 14 factors possessed enough items with loadings exceeding .4 that would also allow for the computation of an internal reliability coefficient (see Fig 1). A subset of ten factors demonstrated an acceptable level of internal reliability, with Cronbach’s alpha values surpassing .7. Consequently, these ten factors (107 items, taking 15–20 minutes to complete) were retained for further analyses without further modification (see Table 4). These ten factors explained 30% of the total variance across the total number of items. We hypothesized that these ten factors would load onto the four theoretically derived functions of *phronesis* as superordinate factors, subject to confirmation in CFA.

**Table 4:** Eigenvalues, internal reliability, and hypothesized function of the ten-factor *phronesis* model.

Factor	Eigenvalue	Number of Items	Cronbach’s Alpha	Example Item	Factor Name	Hypothesized Function
1	19.84	19	.927	I make sure to gather all the details before forming an opinion about what’s right and wrong.	Moral Deliberation	Moral Adjudication
2	7.00	15	.907	I try to consider how my decisions today will reflect on the person I aspire to be in the future.	Aspired Moral Identity	Moral Identity
3	4.80	20	.905	(How able would you be to control your emotions if…) You accidentally spill a drink on your clothes just as you are about to leave the house.	Emotional Regulation	Moral Emotion
4	3.64	6	.829	Consistency between my actions and my values enhances my self-respect.	Moral Self-Relevance	Moral Identity
5	3.33	8	.821	My decisions are influenced by a mix of my thoughts, feelings, and personal beliefs.	Moral Integration	Moral Adjudication
6	2.29	7	.833	(How would you feel about yourself in these scenarios if these were the actions you took. . .) You exaggerate a problem at work to damage a colleague’s professional reputation.	Negative Moral Emotion	Moral Emotion
7	2.10	7	.821	(Select the two most relevant virtues…) Your best friend reveals they cheated on an important exam and asks you to keep it a secret.	Virtue Identification	Moral Perception
8	1.97	9	.762	(How would you feel about yourself in these scenarios if these were the actions you took.?) A stranger drops a £100 note without noticing. You pick it up and return it to them.	Positive Moral Emotion	Moral Emotion
9	1.59	8	.748	(Does your choice in this situation reflect upon your character?) You’re the manager of a restaurant and you catch one of your staff stealing food	Situational Moral Relevance	Moral Perception
10	1.43	8	.725	(Does your choice in this situation reflect upon your character?) You have an opportunity to invest in two promising startups, but you only have funds for one.	Situational Moral Irrelevance	Moral Perception

In naming these ten factors, some item sets contained a mix of general factor items and items that are more directly morally salient. For example, one factor contained items like “I try to consider how my decisions today will reflect on the person I aspire to be in the future”, not specifically mentioning moral aspirations. However, other items in that item set such as “I set personal targets that involve improving my ethical awareness and character” were more directly morally relevant. The fact that all aspirations entail values and conceptions of *the good* notwithstanding, we named factors based on the aggregate meaning of the items constitutive of that factor in the context of the overall measure (e.g., in the case of the two items mentioned above, these were both aspects of “Aspired Moral Identity”). Later in the measure development process, tests of criterion validity could then be used to further establish whether each factor predicts what it should, given how we have named it (see Study 3).

#### Study 1b: Testing the identified dimensions in two new samples using CFA

*Sample 1 (UK)*. Each of the ten factors were once again found to be internally reliable, with Cronbach’s Alphas ranging from .72 to .92. We then used a CFA to assess whether the data would fit the hypothesized ten-factor model. The model converged successfully and we observed excellent model fit (*CFI* = .97, *TLI* = .97; *RMSEA* = .04, *SRMR* = .06; *χ^2^*[5414] = 13178.35, *p* < .001). Next, we used a bifactor structural equation model (SEM) to test whether the ten empirically identified factors could be considered sub-factors of the four theoretical components (Moral Identity, Moral Emotion, Moral Perception, and Moral Adjudication) from which the items were originally derived, and in turn, whether those components loaded onto a superordinate *Phronesis* factor (in what follows, *Phronesis* is capitalized when it refers specifically to the factor in question rather than to the general philosophical concept). The model did not converge after 2,436 iterations, and so reliable estimates for our hypothesized bifactor model in a UK sample could not be produced. We reran this model, this time allowing the ten factors to covary within the hypothesized four factors (e.g., Moral Deliberation allowed to covary with Moral Integration as they are both theoretically aspects of Moral Adjudication), but this model did not converge after 3,749 iterations. The failure to converge suggested potential model misspecification or complexity that the data could not adequately support, limiting our ability to confirm the hypothesized structure. The factor covariances are presented in Table 5 (upper).

**Table 5:** Factor covariance matrices for US and UK samples.

	*1*	*2*	*3*	*4*	*5*	*6*	*7*	*8*	*9*	*10*
**UK Sample**										
**1.** Moral Deliberation	1									
**2.** Aspired Moral Identity	0.341***	1								
**3.** Moral Self Relevance	0.334***	0.431***	1							
**4.** Moral Integration	0.154***	0.202***	0.200***	1						
**5.** Emotional Regulation	0.050***	0.007	0.028*	-0.009	1					
**6.** Virtue Identification	0.005	-0.019	0.025	-0.003	0.017	1				
**7.** Situational Moral Relevance	0.113***	0.091***	0.196***	0.070***	-0.004	0.120***	1			
8. Situational Moral Irrelevance	0.037	-0.064*	0.087**	-0.024	0.019	0.117***	0.180***	1		
**9.** Negative Moral Emotion	0.203***	0.184***	0.314***	0.086***	0.011	0.083***	0.201***	0.147***	1	
**10.** Positive Moral Emotion	0.192***	0.207***	0.197***	0.121***	-0.037*	-0.024	-0.100***	0.031	0.228***	1
**US Sample**										
**1.** Moral Deliberation	**1**									
**2.** Aspired Moral Identity	0.394***	**1**								
**3.** Moral Self Relevance	0.431***	0.481***	**1**							
**4.** Moral Integration	0.196***	0.230***	0.223***	**1**						
**5.** Emotional Regulation	0.062***	0.01	0.056**	-0.025*	**1**					
**6.** Virtue Identification	0.033	0.016	0.085***	0.002	0.038*	**1**				
**7.** Situational Moral Relevance	0.161***	0.130***	0.253***	0.085***	0.038*	-0.168***	**1**			
8. Situational Moral Irrelevance	0.053	-0.039	0.200***	-0.011	0.094***	0.186***	0.250***	**1**		
**9.** Negative Moral Emotion	0.219***	0.197***	0.369***	0.063***	0.065***	0.111***	0.249***	0.263***	**1**	
**10.** Positive Moral Emotion	0.260***	0.228***	0.287***	0.162***	-0.007	0.016	0.186***	0.199***	0.326***	**1**

Note

*p < .05

**p < .01

***p < .001

*Sample 2 (US)*. Consistent with the previous analysis, this CFA was adequately powered, maintaining more than ten participants for each item in the model. We also tested for configural, metric, and scalar invariance. Once again, internal reliability was strong across the ten sub-factors according to Cronbach’s Alpha values which ranged from .72-.92. The model fit for the ten-factor solution in the US sample was also excellent (*CFI* = .97, *TLI* = .97; *RMSEA* = .04, *SRMR* = .07; *χ^2^*[5414] = 15092.52, *p* < .001). A bifactor SEM was then fitted to the data with the ten factors loading onto the four theorized components of *phronesis*, and they in turn onto an overarching *Phronesis* factor. The model did not fully converge. The factor covariances are presented in Table 5 (lower).

*Measurement invariance*. We tested whether the measurement models differed across the US vs UK samples for our best fitting models (i.e., the ten latent factors derived empirically through EFA). The initial step in measurement invariance testing, *configural* invariance, establishes that the factor structure (i.e., the number of factors and their pattern of loadings) is consistent across groups. This configural model served as our baseline for comparison. No constraints are applied to factor loadings or intercepts at this stage. The configural model demonstrated acceptable fit across the two samples (*CFI* = .96, *TLI* = 0.96, *RMSEA* = .03, *SRMR* = .05), indicating that the factor structure was consistent between the US and UK samples. Next, we tested for *metric* invariance by constraining the factor loadings to be equal across groups. The chi-squared difference test between the configural and metric models was significant, *χ^2^*(96) = 589.57, *p* < .001, suggesting a change in fit. This change implies that some factor loadings may differ across groups. Subsequently, *scalar* invariance was assessed by additionally constraining the intercepts to be equal across groups. The chi-squared difference test between the metric and scalar models was also significant, *χ^2^*(96) = 292.48, *p* < .001, indicating that intercepts might also vary across groups. Finally, residual invariance was tested by further constraining the residual variances to be equal across groups. The chi-squared difference test between the scalar and residual models was significant, *χ^2^*(106) = 330.88, *p* < .001, suggesting that the residual variances also differ across the US and UK samples.

#### Study 1c: Test-retest reliability

Internal reliabilities for the retested sample were acceptable (.75-.93). The model fit for a ten-factor structure was also excellent (*CFI* = .99, *TLI* = .99; *RMSEA* = .01, *SRMR* = .06; *χ^2^*[5414] = 5482.13, *p* = .255). Test-retest reliability was established by correlating scores on each of the ten *phronesis* components with scores two months later. The results are presented in Table 6.

**Table 6:** Test-retest reliability of the short *phronesis* measure in a UK-based sample.

Variable	Test-retest Correlation	*p*
Moral deliberation	.51	< .001
Moral integration	.49	< .001
Identity Aspirations	.57	< .001
Moral self-relevance	.60	< .001
Emotional regulation	.70	< .001
Negative moral emotion	.68	< .001
Positive moral emotion	.51	< .001
Virtue identification	.58	< .001
Situational moral relevance	.45	< .001
Situational moral irrelevance	.37	< .001

### Study 1 Discussion

The development of a measure capable of reliably assessing *phronesis*, as articulated by the APM, required a methodological approach that balanced respect for theory with empirical openness. While prior research jumped directly to confirmatory models [19, 65], their aim was to test whether the APM’s theoretical assumptions fit observed data. In contrast, our approach leveraged the APM to guide item development, ensuring construct coverage, but allowed EFA to inductively and impartially determine the number and nature of constructs functionally being measured based on participants’ response patterns. This revealed that the APM’s four-factor structure did not emerge from the data as predicted. Instead, ten distinct and internally reliable factors were identified, suggesting a more complex and nuanced conceptualization of *phronesis* than originally proposed [9, 18]. These findings form the basis of the refined framework we term the *neo*-APM, representing an empirically grounded yet philosophically coherent model of practical wisdom.

The confirmatory phase was then essential for evaluating whether this new conceptualization held up in independent samples. CFA allowed us to test the generalizability of the neo-APM (the theoretical model measured by the SPM) across different populations, moving beyond the exploratory sample to assess whether the ten-factor structure represented a broader phenomenon. By conducting CFAs in two nationally representative samples—one from the UK and one from the US—we were also able to test the universality of the neo-APM across culturally distinct contexts. The results demonstrated excellent fit in both samples, providing robust evidence that the neo-APM captures something enduring and broadly applicable. In contrast, a bifactor model attempting to incorporate the neo-APM’s ten factors within the theoretical framework from which the items were derived (i.e., the APM) was not supported. Testing the neo-APM (as measured using the SPM) in two different countries also revealed subtle differences: for example, factor covariance levels were generally higher in the US sample than in the UK, suggesting that sociocultural or even sociobiological factors might influence the relationships among constructs. These findings underscore the universality of the neo-APM as a theoretical framework while highlighting the flexibility needed to accommodate cultural particularities.

Study 1c extended this validation by examining the measure’s temporal stability, a crucial step in determining whether *phronesis* reflects a stable aspect of one’s character or if it fluctuates across time. Test-retest reliability analyses demonstrated moderate stability overall, with higher stability observed in constructs related to general behavioral tendencies, such as Emotional Regulation. In contrast, performance-based constructs like Situational Moral Irrelevance appeared more influenced by transient states (e.g., mood or fatigue). These findings suggest that *phronesis* exhibits characteristics of a stable character trait but is also responsive to situational factors. In essence, the evidence points to *phronesis* as both enduring and adaptive, anchored in dispositions while remaining affected by the changing demands of life.

The interrelationships among specific factors also offer valuable insights into the nature of *phronesis*. For example, Moral Deliberation was strongly correlated with Aspired Moral Identity and Moral Self-Relevance, suggesting that thoughtful moral reasoning is closely tied to a sense of moral aspiration and the integration of morality into one’s self-concept. However, the lack of covariance between Moral Deliberation and Virtue Identification challenges the assumption that recognizing virtues in specific contexts is a prerequisite for engaging in moral reasoning. This finding prompts a re-evaluation of the importance of virtue literacy in moral/character education, at least in adults (such as our current participants). Similarly, the absence of correlation between Emotional Regulation and Negative Moral Emotion raises pointed questions about their integration under the Moral Emotion dimension of the APM. These results highlight the need to move beyond hierarchical assumptions, suggesting instead that *phronesis* may be better conceptualized as a dynamic network of interdependent capacities. This relational complexity is further explored in Study 2.

The first study (1a-1c) provides a robust foundation for understanding *phronesis*, advancing both its theoretical conceptualization and empirical measurement. The neo-APM represents a model that is not only grounded in Aristotelian philosophy but also refined through empirical analysis. However, for this framework to be truly meaningful, it is not enough for the measure to identify patterns that emerge consistently across populations. Establishing the relative importance of the neo-APM components (measured via the SPM) as part of an interrelated network of factors is key for educational purposes, as educators may wish to focus on the most central components, while researchers with limited assessment time may wish to only measure the most central components. This is addressed in Study 2. A comprehensive validation must also show that these patterns correspond to what we would expect based on the theory. In other words, the measure should connect with external benchmarks in ways that align with the philosophical and conceptual understanding of *phronesis*. Study 3 addresses this critical task by examining whether the SPM produces scores that correlate with outcomes and traits Aristotle might have predicted to be linked to practical wisdom—such as flourishing and moral reasoning—as well as related constructs like personality, which, while not conceptualized in Aristotle’s time, are recognized in modern social science as important factors in understanding human behavior. This step tests whether the constructs measured by the SPM behave as predicted when compared to well-established concepts, ensuring that the measure operates in accordance with its theoretical foundations. By addressing these gaps, Study 3 strengthens the neo-APM as a comprehensive framework for understanding *phronesis* and its broader relevance to human capacities.

## Study 2: A nomological network analysis of Aristotelian *phronesis*

### Study 2 Purpose and aims

Given the nuanced findings from Study 1, which highlighted unexpected factor covariation, there emerges a compelling case for reevaluating our approach to understanding the organizational structure of the *phronesis* components initially derived from the APM. This reconsideration led us to pivot towards a network psychometrics framework for Study 2. Unlike traditional psychometric approaches that prioritize the identification of latent constructs to explain observable phenomena, network psychometrics offers a fundamentally different perspective. Traditional models often grapple with the causal directionality implied by latent constructs, invoking a familiar behaviorist critique: If *phronesis* is considered the cause of its sub-factors, then what causes *phronesis* itself? This line of questioning potentially unravels into an infinite regress, where each supposed cause requires another, deeper cause, *ad infinitum* [66].

Network psychometrics sidesteps this dilemma by positing psychological phenomena as the emergent outcomes of complex interactions among observable variables, analogous to how the brain itself works [42, 43, 67]. In this framework, the focus shifts to the structure of these interactions, with particular attention being paid to the *centrality* of specific variables within the network [68]. Such a perspective suggests that among the ten empirically derived aspects of *phronesis* identified in Study 1a, certain factors may play more pivotal roles than others. For example, these more central factors could act as orienting tele within the *phronesis* network, guiding the system’s overall direction and influence on flourishing. By adopting this nomological network approach in Study 2, we aim to delve deeper into the architecture of *phronesis*, exploring how its components interrelate and which, if any, among them serve as the linchpins in the overall network. We anticipated that this would shed light on the emergent properties of the *phronesis network*, offering a fresh and novel lens through which to view its contribution to flourishing. Moreover, in Study 1b, factor covariances were stronger in the US compared to the UK. Therefore, this study also served as an opportunity to test whether overall *phronesis* network connectivity would be significantly stronger in the US versus the UK.

### Study 2 Method

#### Participants and design

In this exploratory study, we aimed to test the network structure of *Phronesis* (i) in general (USA and UK combined), and (ii) exploring US versus UK differences, using cross-sectional data from Study 1b.

#### Analysis plan

We employed network analysis [69] for this study. The analysis hinged on constructing and examining correlation-based networks using *qgraph* [70] and *igraph* [71] in R. This approach allowed us to map out how various moral and psychological components, such as Moral Deliberation, Identity Aspirations, and Emotional Regulation, are interconnected. For each analysis we computed key centrality measures: Betweenness, Closeness, Strength (or Degree), and Expected Influence. These are defined as follows:

*Betweenness*. This is a measure of centrality in a network, reflecting the extent to which a node (i.e., one of the ten *phronesis* factors, in this case) lies on the shortest path between other nodes. Nodes with high betweenness centrality can be seen as important intermediaries or “bridges” in the network, facilitating communication or interaction between different parts of the network.

*Closeness*. Closeness centrality measures how close a node is to all other nodes in the network, based on the shortest paths that connect them. A node with high closeness centrality can quickly interact with all others, indicating it has a central position in the network’s overall structure.

*Strength*. In the context of weighted networks, the strength of a node is the sum of the weights of the edges connected to it. For psychological networks such as these, this can be interpreted as the overall level of direct influence a node has within the network, taking into account the strength of its connections to other nodes.

*Expected influence*. Expected influence is a measure adapted from strength for networks that include both positive and negative edge weights. It sums up the edge weights while considering the sign of each edge, providing a measure of a node’s total positive *or* negative influence on the network. A high positive expected influence indicates a node’s strong positive impact on connected nodes, whereas a high negative value suggests a strong negative impact.

All network analyses used the Extended Bayesian Information Criterion (EBIC) using the *glasso* [72] and *bootnet* [73] packages to penalize for model complexity. The EBIC helps in selecting a model that is parsimonious yet sufficiently explanatory, thereby preventing overfitting and ensuring the model’s generalizability. This EBICglasso method performs thresholding by penalizing small partial correlations towards zero. This results in a network where only statistically robust and meaningful connections are retained.

### Study 2 Results

#### Overall

**Fig 2:**
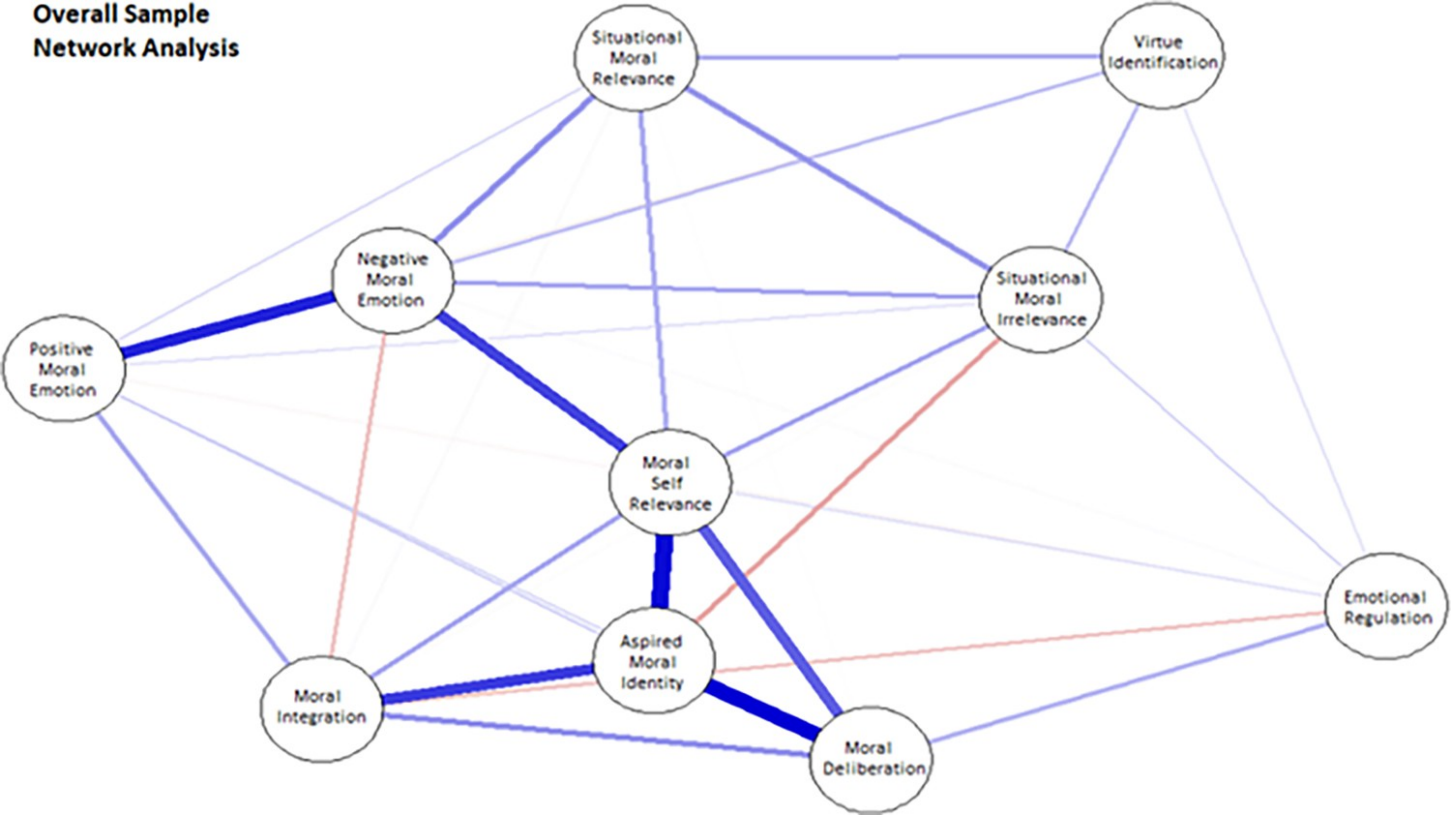
An overall *phronesis* network model. Note. EBIC penalization applied.

In Table 7, we present the measures of centrality for each component within the *phronesis* network, with the network’s overall structure depicted in Fig 2. These centrality statistics help us understand the relative importance or influence of each component within the network. For instance, Betweenness highlights components that act as central connectors or bridges. Moral Self-Relevance, with a high Betweenness score of 26, serves as a key link between otherwise disconnected parts of the network, whereas components like Emotional Regulation and Virtue Identification, both with Betweenness scores of 0, indicate a more isolated position. Closeness reveals how central a component is by measuring its proximity to others in the network. In this context, Moral Self-Relevance (Closeness: 0.0137) is more centrally located compared to Virtue Identification (Closeness: 0.0067), meaning it has a broader reach within the network. Strength reflects the weight of a component’s direct connections. For example, Moral Self-Relevance has the highest Strength score (1.212), indicating strong and numerous connections, while Emotional Regulation and Virtue Identification have weaker connections (Strength: 0.351). Finally, Expected Influence considers the potential impact of a component, accounting for both the strength and direction of its connections. Identity Aspirations and Moral Self-Relevance (Expected Influence: 0.929 and 0.928, respectively) have significant potential to shape the network, whereas Emotional Regulation (Expected Influence: 0.138) plays a more limited role. The range of these statistics—from highly positive (e.g., 1.212 in Moral Self-Relevance’s Strength) to positive but less influential (e.g., 0.138 in Emotional Regulation’s Expected Influence)—helps delineate the spectrum of influence within the *phronesis* network, from central and pivotal to isolated or less impactful components.

**Table 7:** Centrality statistics for the overall *phronesis* network model.

	Betweenness	Closeness	Strength	Expected influence
Moral Deliberation	8	0.0113	0.891	0.891
Identity Aspirations	14	0.0128	1.164	0.929
Moral Self-relevance	26	0.0137	1.212	0.928
Moral Integration	2	0.0101	0.832	0.645
Emotional Regulation	0	0.0063	0.351	0.138
Virtue Identification	0	0.0067	0.351	0.284
Sit. Moral Relevance	0	0.0092	0.565	0.565
Sit. Moral Irrelevance	0	0.0100	0.673	0.673
Neg. Moral Emotion	20	0.0128	1.009	0.840
Pos. Moral Emotion	0	0.0092	0.681	0.564

Note. Closeness values are multiplied by 10^−3^ for clarity.

Notably, five components emerge as particularly central within this network: Negative Moral Emotion, Moral Deliberation, Aspired Moral Identity, Moral Self Relevance, and Moral Integration. These central components, highlighted by the bold paths in Fig 2 (blue indicates positive relationships, red indicates negative relationships), play pivotal roles in the network, indicating that their influence is more coordinated and foundational to the overall network conceptualization of *phronesis*. The centrality of these components signifies not only their individual importance but also their collective contribution to the network’s coherence and functionality. This distinction between central and less-central factors is pivotal, as it underscores the varying degrees of influence different components exert within the *phronesis* network. Understanding these dynamics offers insights into how individual components interconnect to form a relatively comprehensive model of moral and ethical reasoning.

#### US vs UK

**Fig 3:**
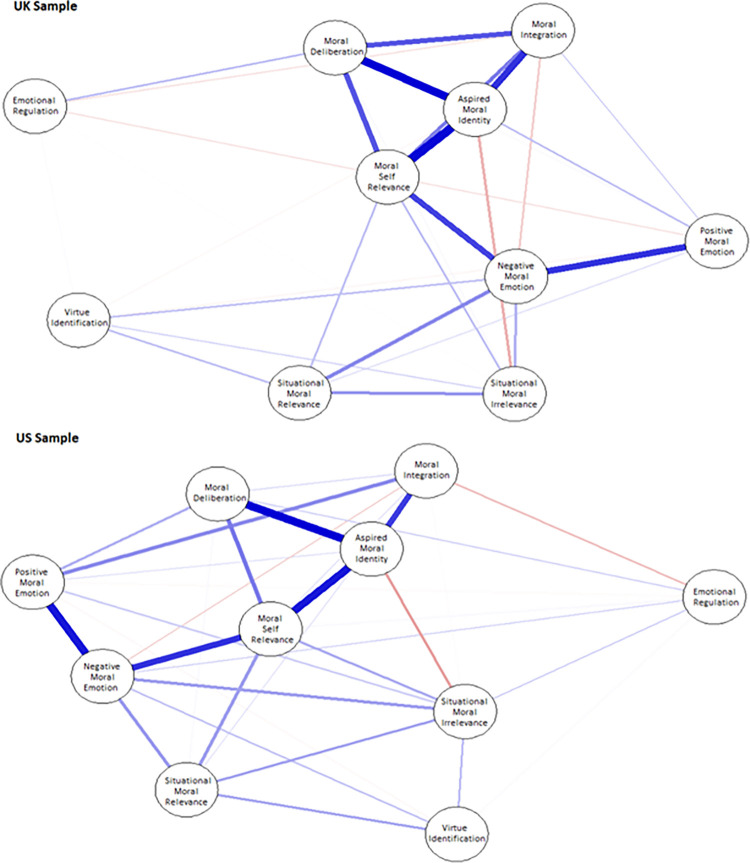
Network plots for the US vs UK *phronesis* network models. Note. EBIC Penalization applied.

Next, we explored whether the network structure looked any different when separated into UK vs US samples. We found that the same network structure broadly held independent of the sample, with the same four components being central overall. The Moral Integration variable appeared to be less central to the *Phronesis* network in the US compared to the UK, however. Network centrality measures are presented in Table 8, and network plots are presented in Fig 3.

**Table 8:** Centrality statistics for the US vs UK *phronesis* network models.

	Betweenness		Closeness		Strength		Expected influence	
	**US**	**UK**	**US**	**UK**	**US**	**UK**	**US**	**UK**
Moral Deliberation	0	16	0.0107	0.0108	0.779	0.916	0.779	0.916
Identity Aspirations	12	6	0.0128	0.0119	1.123	1.158	0.868	0.911
Moral Self-relevance	16	32	0.0131	0.0126	1.101	1.142	1.101	1.142
Moral Integration	6	0	0.0108	0.0095	0.703	0.834	0.390	0.622
Emotional Regulation	0	0	0.0062	0.0055	0.344	0.187	0.131	0.022
Virtue Identification	0	0	0.0065	0.0060	0.335	0.289	0.279	0.177
Sit. Moral Relevance	6	0	0.0098	0.0085	0.615	0.493	0.615	0.493
Sit. Moral Irrelevance	4	0	0.0109	0.0082	0.729	0.496	0.729	0.497
Neg. Moral Emotion	12	36	0.0131	0.0122	1.078	0.917	0.959	0.788
Pos. Moral Emotion	4	0	0.0111	0.0090	0.789	0.537	0.693	0.394

Note. Closeness values are multiplied by 10^−3^ for clarity.

We then compared US vs UK *phronesis* networks using the *NetworkComparisonTest* package [74] in R. A network invariance test, encompassing the pattern of connections and the strength of these connections between nodes, suggested a non-significant difference between US and UK network structures (*M* = .12, *p* = .102). We also conducted a global strength invariance test. We found that the overall connectivity of the *phronesis* network was not different in the UK (3.05) compared to the US (3.85); *S* = 0.80, *p* = .094, consistent with covariance matrices observed in Study 1b.

### Study 2 Discussion

Before this study, we developed a set of items inspired by the APM, empirically discovered that they organize into ten factors, verified this ten-factor structure in two nationally representative samples, discovered that these factors are relatively stable over time, and subsequently discovered that the interrelationships between these ten factors could not be predicted by the APM. This left us with a theoretical lacuna whereby we did not know which of these ten factors were most important, nor how they dynamically configured. Therefore, this study adopted an alternative psychometric approach aimed at organizing the ten factors using exploratory network analyses. What stood out from the results of this study was that the most central variables of the *phronesis* network seemed to exclude Emotional Regulation and Virtue Identification, with Situational Moral Relevance and Situational Moral Irrelevance somewhat peripheral to the overall *phronesis* network.

The *phronesis* network was broadly similar in the US compared to the UK. For character educators, this might suggest that interrelationships between network nodes could be leveraged to promote more effective moral learning. For example, The Gulliford-Roberts Hypothesis [75] proposes that there is a unity of virtue such that moral education in relation to a given virtue could have positive knock-on effects for closely related virtues (e.g., justice and truthfulness are both put forth as kinds of intelligent caring). Analogously, it is plausible that there will be a knock-on effect whereby character education targeting a particular *phronesis* component could have a positive effect on related nodes in the network. Moreover, if trying to educate for a particular node in the *phronesis* network, it could be too complex to influence directly. Aspired Moral Identity, for example, is quite an abstract construct that likely requires a holistic educational or therapeutic approach. In this way, we might look to related nodes in the network such that we can triangulate around the construct of interest through influencing related nodes. Let us take Aspired Moral Identity as an example of a complex and abstract construct in the *phronesis* network that we might wish to target with an intervention. Aspired Moral Identity is unlikely to be high unless Moral Self Relevance has been established. In turn, Moral Self Relevance might be established through a discussion with a teacher, parent, coach, or clinician focusing on Positive and Negative Moral Emotion where much more concrete discussion of direct experience can be had (e.g., “How would you feel in this situation? What does that say about your values and the kind of person you want to be?”). While we cannot make causal claims at this stage about which factors are downstream of which, we can generate plausible empirically driven hypotheses about how to leverage network dynamics in character education, specifically within practical wisdom coaching and education.

## Study 3: Criterion validity

### Study 3 Purpose and aims

In this study, we focus on evaluating the criterion validity of the SPM. Criterion validity is essential in psychometric evaluation as it determines whether a measure accurately predicts theoretically relevant outcomes, which is a core reason for developing any psychometric measure. In Study 1, we developed a concise measure of *phronesis* grounded in the APM, balancing theoretical alignment with practical considerations like brevity and ease of use to ensure participant engagement and scoring efficiency. While no measure can capture every nuance of a complex construct, we believe our item set is sufficiently comprehensive to represent *phronesis* effectively. To further establish the validity of the measure, we employ several statistical approaches in Study 3. Correlations are used to assess convergent validity (how well the measure aligns with similar constructs) and discriminant validity (how distinct it is from unrelated constructs) across a variety of measures. Regressions are applied to evaluate predictive validity, determining whether the measure can forecast relevant outcomes over a two-month period. Finally, hierarchical regressions are conducted to assess incremental criterion validity, testing whether the measure explains unique variance in outcomes beyond other established predictors.

Incremental criterion validity is especially important because it demonstrates that the measure captures something distinct and meaningful about the individual that is not already accounted for by other variables or measures. In the context of the SPM, this means that the measure adds unique value in terms of explaining variance in relevant outcomes, showing that *phronesis* is not just an overlap with other constructs we already know about. By explaining additional variance in outcome variables—variables that themselves are important for understanding human flourishing—incremental criterion validity is a clear marker of the unique contribution of the SPM. If the SPM can predict outcomes of interest that other measures cannot, this strengthens the argument that it captures an important and under-explored dimension of a person’s character. Ultimately, these analyses provide a comprehensive evaluation of the measure’s psychometric soundness. Strong evidence for convergent, discriminant, predictive, and incremental criterion validity would affirm the robustness of the SPM, ensuring that it not only aligns with theoretical expectations but also offers unique insights into relevant outcomes.

Now we consider the outcomes sought. The earlier attempt to measure *phronesis* [19] focused on measures that were drawn primarily from the developmental sciences and it had, as a secondary mission, the aim of assessing whether *phronesis* or its components might better address the judgment–action gap, which is a central controversy in the field [2]. Thus, the outcome of interest used in the earlier work by the current research center was prosocial action. In the current study, we chose to broaden the focus and assess flourishing as the main outcome of interest [7, 10]. As described in the introduction, we hypothesized that *phronesis* is associated with an increased likelihood of flourishing, or the sense that life in its more central dimensions (not merely “subjective wellbeing”, but also other aspects of flourishing) is seen in a positive light. We also correlate *phronesis* with personality and a host of morally salient variables to help situate *phronesis* empirically amongst other established psychological constructs. Finally, to help to establish that *phronesis*, as measured here, has practical implications over and above other major psycho-moral theories, we tested whether *phronesis* predicts flourishing over and above the most recent and comprehensive measure emerging from Moral Foundations Theory [36].

### Study 3 Methods

#### Participants

The two samples first described in Study 1b were included here. As described earlier, one sample was collected in the UK and a second in the USA. The UK sample included 1,000 participants with 997 retained after removing participants who wished to withdraw. Sample 2, collected in the US, included 1,000 participants, with 988 retained after removing participants who wished to withdraw. Furthermore, we tested the degree to which *phronesis* predicted flourishing over a two-month period with the 300-person sub-sample from Study 1c. The aggregated demographics for the two samples are represented in Table 9 below:

**Table 9:** Demographics for the combined UK/US sample.

Age	*n(%)*	Ethnicity	*n(%)*	Sex	*n(%)*
18–25	262(13.23)	Arab	5(.25)	Female	1009(50.96)
26–35	351(17.73)	Black	159(8.03)	Male	951(48.03)
36–45	370(18.69)	Central Asian	44(2.22)	Prefer not to say	5(.25)
46–55	332(16.77)	East Asian	68(3.43)	Self-identify	15(.76)
56–65	428(21.62)	Mixed/ multiple ethnic groups	50(2.53)		
66–75	211(10.66)	Prefer not to say	12(.61)		
76–85	22(1.11)	White	1636(82.63)		
Other	4(.20)				

#### Procedure

Following an information page, participants were requested to give their consent to participation. Participants in both samples received a battery of assessments (details below). A subset of 300 UK participants were measured two months later using the SPM and a flourishing measure (see below). After completing the questionnaires, participants were given a debriefing and expression of gratitude for their involvement.

#### Measures

*Phronesis*. The full set of *phronesis* measurement items described in Study 1b was included in these analyses. This measure was administered to both the UK and US samples.

*Flourishing*. We also included in the analysis the WBA [59]. As described in Study 1a, this instrument is designed to gauge comprehensive wellbeing following the World Health Organization’s holistic definition of health, which includes mental, physical, and overall wellbeing. Specifically, the WBA assesses six domains: emotional health, physical health, meaning and purpose, character strengths, social connectedness, and financial stability. This stands as a reasonable proxy for Aristotle’s notion of flourishing updated for the current era [46, 76]. This measure was administered to both the UK and US samples.

*HEXACO-PI-R*. Both US and UK participants completed the HEXACO-PI-R [77] to measure the Big Six personality traits, and their various sub-factors:

1. **Honesty-Humility**: Measures levels of sincerity, fairness, greed-avoidance, and modesty.
2. **Emotionality**: Captures attributes such as fearfulness, anxiety, dependence, and sentimentality.
3. **Extraversion**: Focuses on social self-esteem, social boldness, sociability, and liveliness.
4. **Agreeableness**: Incorporates qualities like forgiveness, gentleness, flexibility, and patience.
5. **Conscientiousness**: Evaluates organization, diligence, perfectionism, and prudence.
6. **Openness to Experience**: Explores facets like aesthetic appreciation, inquisitiveness, creativity, and unconventionality.

This measure was administered to both the UK and US samples.

*The dark tetrad*. We measured the four “dark” personality traits (psychopathy, sadism, Machiavellianism, and narcissism) using the SD4 [78] in both US and UK participants. This was a 28-item measure to which participants responded to statements about themselves on a Likert scale from Strongly disagree–Strongly agree. This measure was administered to both the UK and US samples.

*The moral foundations questionnaire*. The MFQ2 was used to assess Moral Foundations, *qua* Moral Foundations Theory (MFT; see [36]). It measures the core moral dimensions according to MFT, namely, Care, Authority, Loyalty, Purity, Proportionality, and Equality. This was administered to the UK-representative sample only.

*Propensity to morally disengage*. The PMDS [79] is a 16-item measure aims to gauge the extent to which participants exhibited a willingness to engage in immoral actions. This scale is comprised of 16 items that span eight distinct factors. These factors are:

*Distortion of consequences*. For example, the item “Taking personal credit for ideas that were not your own is no big deal” probes this aspect.

*Dehumanization*. Illustrated by the item “Some people have to be treated roughly because they lack feelings that can be hurt.”

*Attribution of blame*. Captured by items like “People who get mistreated have usually done something to bring it on themselves.”

*Diffusion of responsibility*. For instance, the item “It’s okay to tell a lie if the group agrees that it’s the best way to handle the situation.”

*Displacement of responsibility*. Examined by the item “People shouldn’t be held accountable for doing questionable things when they were just doing what an authority figure told them to do.”

*Moral justification*. Represented by the item “It is alright to lie to keep your friends out of trouble.”

*Advantageous comparisons*. For example, the item “Considering the ways people grossly misrepresent themselves, it’s hardly a sin to inflate your own credentials a bit.”

*Euphemistic labelling*. As per the item “It’s okay to gloss over certain facts to make your point.”

Participants responded to these items using a seven-point Likert scale ranging from “Strongly Disagree” to “Strongly Agree.” This framework allows for a multi-dimensional exploration of an individual’s propensity towards moral disengagement, thereby providing a comprehensive understanding of various aspects that contribute to immoral decision-making. This was administered to the UK-representative sample only.

#### Analytical strategy

Simple Spearman correlation matrices were used to explore linear relations between *phronesis* and (i) flourishing sub-factors, (ii) Big Six personality traits and dark personality traits, (iii) moral disengagement factors, and (iv) Moral Foundations. This was important to show convergent and discriminant validity of the SPM (i.e., that it performs empirically as might be predicted theoretically in relation to other established measures). For these analyses we used the *ggplot2* [80], *GGally* [81], and *ggcorrplot* [82] packages in R. We also used linear regressions to predict flourishing components using *phronesis* network components over a two-month interval to establish the predictive validity of the SPM, going above and beyond simple correlations. Finally, we used multiple linear regression to predict various outcomes of interest using *phronesis* network components, over and above moral foundations. This allowed us to test whether the SPM can explain variance in outcomes of interest over and above one of the most popular measures found within the moral psychology domain, thereby establishing that the SPM measures something important (insofar as the outcome variables are important) that the MFQ2 does not. All analyses were conducted in R Studio [62] using aforementioned packages, or using Jamovi [83] with an R [84] backend. The *car* package [85] was used to perform hierarchical regression analyses within Jamovi to establish incremental criterion validity of the *phronesis* network components over and above moral foundations.

### Study 3 Results

#### Flourishing

We first explored whether *phronesis* components correlated with flourishing components (see Table 10). The same general pattern and magnitude of correlations was found within both samples. These findings suggest that all aspects of *phronesis* aside from the perceptual components (Virtue Identification, Situational Moral Relevance, and Situational Moral Irrelevance) correlated with all aspects of flourishing. The strongest correlations, as might be expected, were between *phronesis* components and Character Strengths, while the weakest correlations were between *phronesis* components and Financial Security.

**Table 10:** Correlation matrices including WBA flourishing factors alongside ten components of *phronesis* in the UK (upper) and US (lower) samples.

Phronesis Component	Emotional Health	Physical Health	Social Connectedness	Character Strengths	Meaning and Purpose	Financial Security
UK Sample						
Virtue Identification	0.03	0.00	0.01	0.03	0.05	0.04
Situational Moral Relevance	0.01	0.02	0.03	0.01	-0.01	-0.02
Situational Moral Irrelevance	-0.03	-0.01	0.00	-0.02	0.02	-0.04
Emotional Regulation	0.21***	0.15***	0.14***	0.23***	0.14***	0.01
Negative Moral Emotion	0.11***	0.10**	0.18***	0.30***	0.23***	0.06
Positive Moral Emotion	0.13***	0.11***	0.15***	0.29***	0.19***	-0.01
Moral Deliberation	0.17***	0.16***	0.18***	0.39***	0.27***	0.07*
Moral Integration	0.08*	0.06	0.12***	0.15***	0.14***	0.00
Moral Self Relevance	0.12***	0.10**	0.17***	0.32***	0.24***	0.03
US Sample						
Virtue Identification	0.03	0.06	0.06	0.06	0.07*	-0.03
Situational Moral Relevance	0.01	0.02	0.03	0.09**	0.06	0.03
Situational Moral Irrelevance	-0.06	-0.03	-0.06	-0.06	-0.04	-0.17***
Emotional Regulation	0.20***	0.16***	0.17***	0.19***	0.17***	0.09**
Negative Moral Emotion	0.19***	0.08*	0.22***	0.34***	0.28***	0.05
Positive Moral Emotion	0.10**	0.09**	0.16***	0.24***	0.14***	-0.01
Moral Deliberation	0.20***	0.14***	0.18***	0.45***	0.28***	0.08*
Moral Integration	0.03	0.02	0.01	0.08*	0.04	0.00

Note

*p < .05

**p < .01

***p < .001

*Phronesis* components were collectively entered as predictors within a linear regression, with flourishing variables measured two months later as the outcome variables. *Phronesis* components collectively functioned as strong predictors of all aspects of flourishing two months later apart from Financial Security. The findings are found in Table 11. Additionally, baseline *phronesis* predicted Emotional Health two months later over and above baseline Emotional Health (Model 1 *r*^*2*^ = .47, Model 2 *r*^*2*^ = .51; *F*[10, 283] = 2.27, *p* = .014).

**Table 11:** Predicting flourishing using the ten *phronesis* components two months later.

Outcome	*R*	*R^2^*	Adjusted *R^2^*	*F*	df1	df2	*p*
Emotional Health	.406	.165	.136	5.59	10	283	< .001
Physical Health	.339	.115	.084	3.68	10	283	< .001
Meaning and Purpose	.403	.162	.133	5.48	10	283	< .001
Financial Security	.248	.062	.029	1.86	10	283	.051
Social Connectedness	.423	.179	.150	6.16	10	283	< .001
Character Strengths	.584	.341	.318	14.6	10	283	< .001

#### Flourishing and the *phronesis* network

**Fig 4:**
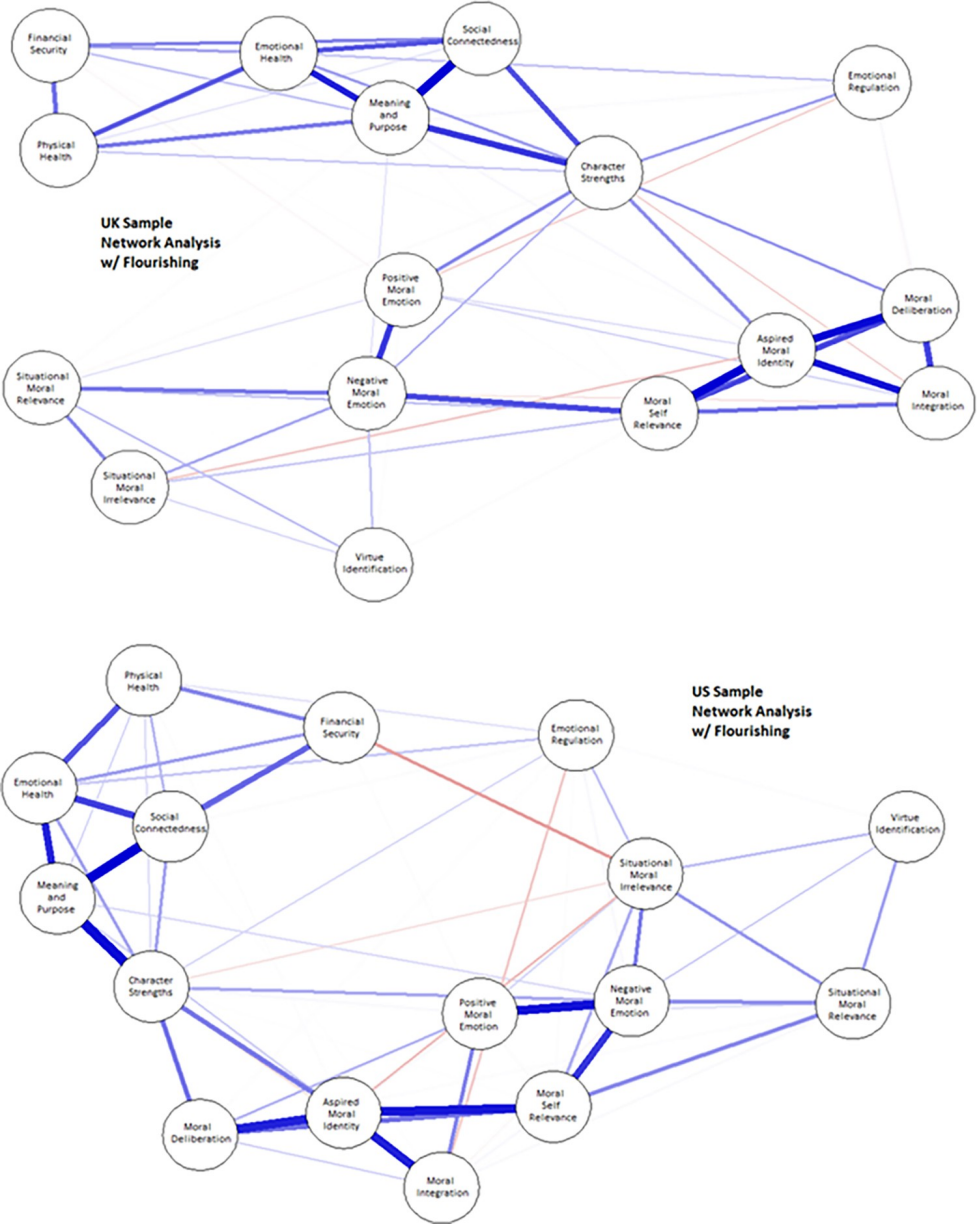
Flourishing in the context of the *phronesis* network in UK (upper) vs US (lower) samples. Note. EBIC penalization applied.

At the next step, flourishing variables from the WBA were introduced alongside the SPM *phronesis* network variables for both the UK and US samples. In previous network models, the four most important aspects of the overall *Phronesis* network were adjudicative variables (namely Moral Deliberation and Moral Integration) and identity variables (namely, Moral Self-relevance and Aspired Moral Identity). Network analyses presented below suggest that Moral Integration is more on the periphery of the *phronesis* network among US participants compared to UK participants. Moreover, these network models appear to suggest that Social Connectedness is central to overall flourishing, with Financial Security only peripherally related to the *phronesis* network compared to other flourishing variables. In both the US and the UK, Emotional Regulation sits somewhat separate from the other *phronesis* and flourishing variables, but it nonetheless supports both. For both samples, Meaning and Purpose as well as Character Strengths were the most closely related aspects of flourishing to the *phronesis* network, as might have been expected theoretically. A network invariance test suggested a non-significant difference between US and UK network structures (*M* = .10, *p* = .408). As before, we also conducted a global strength invariance test. We found that the overall connectivity of the *phronesis* network with flourishing was no different in the USA (6.34) compared to the UK (5.72); *S* = .62, *p* = .249. These relationships between variables in both networks/samples are plotted in Fig 4 and centrality statistics are tabulated in Table 12.

**Table 12:** Centrality statistics for the US vs UK *phronesis* network models in the context of flourishing.

Phronesis Component	Betweenness		Closeness		Strength		Expected influence	
	US	UK	US	UK	US	UK	US	UK
Moral Deliberation	0	0	0.00537	0.00495	0.792	0.889	0.792	0.889
Identity Aspirations	44	32	0.00593	0.00531	1.172	1.183	1.022	1.022
Moral Self-relevance	28	24	0.00556	0.00497	1.003	1.082	1.003	1.072
Moral Integration	10	0	0.00491	0.00431	0.681	0.856	0.371	0.619
Emotional Regulation	0	0	0.00328	0.00336	0.347	0.317	0.217	0.123
Virtue Identification	0	0	0.00301	0.00251	0.255	0.214	0.255	0.161
Situational Moral Relevance	12	0	0.00437	0.00368	0.568	0.505	0.568	0.446
Situational Moral Irrelevance	28	0	0.00502	0.00307	0.791	0.407	0.333	0.273
Negative Moral Emotion	30	74	0.00552	0.00505	1.085	0.922	1.029	0.843
Positive Moral Emotion	4	8	0.00459	0.00499	0.687	0.605	0.668	0.407
Emotional Health	24	2	0.00469	0.00413	0.997	0.949	0.997	0.949
Physical Health	0	0	0.00390	0.00359	0.590	0.631	0.590	0.631
Social Connectedness	12	24	0.00488	0.00470	0.965	0.897	0.954	0.897
Character Strengths	60	104	0.00563	0.00574	1.153	1.262	0.996	1.113
Meaning and Purpose	52	42	0.00533	0.00490	1.157	1.129	1.126	1.103
Financial Security	18	0	0.00450	0.00336	0.630	0.544	0.630	0.428

#### HEXACO personality and the dark tetrad

We next sought to explore the personality correlates of *phronesis*. This included both the Big 6 and the Dark Tetrad (see Table 13). The general pattern of correlations between *phronesis* components and personality was positive. Notably, the three strongest correlations were between Conscientiousness and (i) Moral Deliberation, (ii) Moral Self Relevance, and Negative Moral Emotion, three of the most central components of the *phronesis* network. Emotional Regulation was moderately negatively related to trait Negative Emotion, as might be expected. Negative Moral Emotion was only slightly positively related to the general personality trait of Negative Emotion, indicating they are not the same thing. The general pattern of correlations shows negative associations between *phronesis* components and the Dark Tetrad. The main exception to this was that those with higher Aspired Moral Identity and Moral Integration were slightly more likely to be Narcissistic. Situational Moral Relevance, Situational Moral Irrelevance, and Virtue Identification, our three objective measures of moral perception, were shown to have criterion validity here as they were negatively associated with the Dark Tetrad, on the whole. As might be expected, the strongest negative relationships were between the moral perception variables and the main trait associated with moral perceptual deficits–Psychopathy.

**Table 13:** Correlation matrix showing the relationships between phronesis network components and personality (HEXACO and dark tetrad).

Phronesis Component	Honesty-Humility	Negative Emotion	Extraversion	Agreeableness	Conscientiousness	Openness	Machiavellianism	Narcissism	Psychopathy	Sadism
Virtue Identification	0.10***	-0.01	0.03	0.06**	0.14***	0.05*	-0.03	-0.01	-0.10***	-0.08***
Situational Moral Relevance	0.15***	0.07**	-0.02	0.05*	0.15***	0.10***	-0.04	-0.09***	-0.15***	-0.13***
Situational Moral Irrelevance	0.20***	0.01	-0.04	0.04	0.16***	0.09***	-0.09***	-0.17***	-0.25***	-0.15***
Emotional Regulation	0.15***	-0.32***	0.19***	0.33***	0.18***	0.14***	-0.12***	0.04	-0.07**	-0.07**
Negative Moral Emotion	0.51***	0.14***	0.13***	0.28***	0.36***	0.18***	-0.27***	-0.23***	-0.44***	-0.48***
Positive Moral Emotion	0.13***	0.14***	0.07**	0.13***	0.19***	0.10***	0.00	-0.04*	-0.21***	-0.12***
Moral Deliberation	0.20***	-0.05*	0.18***	0.26***	0.39***	0.23***	-0.03	0.03	-0.18***	-0.12***
Moral Integration	-0.06*	0.18***	0.02	0.02	0.04	0.11***	0.10***	0.05*	-0.02	0.01
Moral Self-Relevance	0.30***	0.08***	0.13***	0.19***	0.34***	0.25***	-0.12***	-0.05*	-0.21***	-0.21***
Aspired Moral Identity	0.08***	0.08***	0.24***	0.24***	0.25***	0.14***	-0.03	0.16***	-0.06**	-0.08***

Note

*p < .05

**p < .01

***p < .001

#### Moral disengagement

Given the complex pattern of correlations between *phronesis* and personality, including the dark personality traits, it was important to explore the relationship between *phronesis* and morally salient variables in more detail. To that end, we next correlated *phronesis* components with moral disengagement variables (see Table 14). The results showed a much clearer picture whereby all *phronesis* components were negatively related to different indices of the propensity to morally disengage, creating a much clearer picture of criterion validity.

**Table 14:** Correlations between *phronesis* network components and propensity to morally disengage.

Phronesis Component	Moral Justification	Euphemistic Labelling	Advantageous Comparison	Displacement of Responsibility	Diffusion of Responsibility	Distortion of Consequences	Dehumanization	Attribution of Blame
Virtue Identification	-0.08**	-0.03	-0.13***	-0.05	-0.11***	-0.09**	-0.11***	-0.10**
Moral Relevance	-0.06	-0.09**	-0.13***	-0.11***	-0.18***	-0.17***	-0.13***	-0.18***
Moral Irrelevance	-0.07*	-0.03	-0.11***	-0.08*	-0.11***	-0.11***	-0.14***	-0.15***
Emotional Regulation	-0.04	-0.04	-0.05	-0.05	-0.02	-0.03	-0.12***	-0.09**
Negative Moral Emotion	-0.49***	-0.43***	-0.56***	-0.31***	-0.48***	-0.56***	-0.43***	-0.52***
Positive Moral Emotion	-0.06	-0.10**	-0.23***	-0.14***	-0.20***	-0.17***	-0.06	-0.15***
Moral Deliberation	-0.13***	-0.15***	-0.19***	-0.15***	-0.17***	-0.16***	-0.10**	-0.13***
Moral Integration	0.08**	0.01	-0.03	-0.02	-0.01	-0.02	0.05	-0.03
Moral Self-Relevance	-0.17***	-0.19***	-0.26***	-0.18***	-0.22***	-0.25***	-0.16***	-0.24***
Aspired Moral Identity	-0.09**	-0.15***	-0.13***	-0.05	-0.10**	-0.15***	-0.06	-0.08**

Note

*p < .05

**p < .01

***p < .001

#### Moral foundations

The concept of *phronesis* emphasizes a deliberate and rational approach to moral decision making, in the service of flourishing. However, other moral theories emphasize the role of foundational emotional intuitions, driven by mostly innate sentiments, in moral judgements. Arguably the most well-studied sentimentalist moral theory is Moral Foundations Theory, which emphasizes Purity, Authority, Loyalty, Proportionality, Equality, and Care as the basic moral intuitions [36]. As a non-sentimentalist moral construct, but a moral construct nonetheless, we would therefore expect some degree of correlation between Moral Foundations and *phronesis*. When we tested this (see Table 15), we found that several aspects of the *phronesis* network were positively related to Moral Foundations. Notably though, the Emotional Regulation and Situational Moral Irrelevance aspects of the *phronesis* network were negatively related to Moral Foundations, reflecting the non-sentimentalist emphasis within *phronesis*.

**Table 15:** Correlations between *phronesis* network components and moral foundations.

Phronesis Component	Care	Equality	Proportionality	Loyalty	Authority	Purity
Virtue Identification	0.02	-0.06	0.04	-0.02	0.03	-0.05
Situational Moral Relevance	0.19***	0.03	0.05	-0.04	-0.09**	-0.09**
Situational Moral Irrelevance	0.03	-0.09**	-0.01	-0.13***	-0.13***	-0.18***
Emotional Regulation	-0.02	-0.04	-0.05	-0.03	-0.07*	-0.08**
Negative Moral Emotion	0.37***	0.02	0.07*	0.10**	0.13***	0.05
Positive Moral Emotion	0.34***	0.07*	0.29***	0.16***	0.21***	0.08*
Moral Deliberation	0.25***	0.09**	0.15***	0.12***	0.10**	0.09**
Moral Integration	0.19***	0.10**	0.19***	0.09**	0.10**	0.09**
Moral Self-Relevance	0.32***	0.03	0.13***	0.04	0.02	0.03
Aspired Moral Identity	0.34***	0.15***	0.21***	0.21***	0.19***	0.25***

Note

*p < .05

**p < .01

***p < .001

Given the mixed associations between *phronesis* network components and Moral Foundations, we then decided to test which set of constructs best predicts our key criterion valiables–the flourishing network. Therefore, we used a hierarchical linear regression to test—whether the network of variables constitutive of *phronesis* would predict aspects of flourishing over and above Moral Foundations. We first entered moral foundations components (Care, Equality, Proportionality, Loyalty, Authority, Purity) as the Model 1 predictors, and *phronesis* network components as Model 2 predictors. Model comparison results are found in Table 16, and full beta coefficients are found in the supplementary file “MFT_comparisons”. Table 16 shows that moral foundations explain between 5% (Emotional and Physical Health) and 24% (Character Strengths) of the variance in flourishing outcomes. When using *phronesis* network components to predict these flourishing outcomes over and above Moral Foundations, *phronesis* network components explain large amounts of additional variance in all flourishing oucomes except Financial Security, ranging between +5% (Physical Health) to +18% (Character Strengths).

**Table 16:** Predicting outcomes of interest with *phronesis* network components, over and above moral foundations.

Outcome	Model 1 Adj R^2^	Model 1 Sig	Model 2 Adj R^2^	ΔR^2^	ΔR^2^ Sig
Emotional Health	0.05	*F*(6, 988) = 10.56, *p* < .001	0.12	0.07	*F*(10, 978) = 8.33, *p* < .001
Physical Health	0.05	*F*(6, 988) = 8.87, *p* < .001	0.1	0.05	*F*(10, 978) = 5.79, *p* < .001
Social Connectedness	0.1	*F*(6, 988) = 19.10, *p* < .001	0.15	0.06	*F*(10, 978) = 6.51, *p* < .001
Financial Security	0.09	*F*(6, 988) = 18.25, *p* < .001	0.1	0.01	*F*(10, 978) = 1.28, *p* = .240
Meaning and Purpose	0.13	*F*(6, 988) = 25.00, *p* < .001	0.21	0.09	*F*(10, 978) = 11.70, *p* < .001
Character Strengths	0.24	*F*(6, 988) = 53.20, *p* < .001	0.42	0.18	*F*(10, 978) = 30.90, *p* < .001
Honesty/Humility	0.14	*F*(6, 987) = 27.20, *p* < .001	0.23	0.1	*F*(10, 977) = 12.90, *p* < .001
Machiavellianism	0.06	*F*(6, 987) = 12.10, *p* < .001	0.15	0.09	*F*(10, 977) = 10.80, *p* < .001
Narcissism	0.05	*F*(6, 987) = 10.20, *p* < .001	0.17	0.13	*F*(10, 977) = 15.40, *p* < .001
Psychopathy	0.06	*F*(6, 987) = 11.60, *p* < .001	0.26	0.2	*F*(10, 977) = 26.80, *p* < .001
Sadism	0.12	*F*(6, 987) = 23.40, *p* < .001	0.35	0.23	*F*(10, 977) = 34.90, *p* < .001
Moral Justification	0.07	*F*(6, 987) = 14.10, *p* < .001	0.33	0.26	*F*(10, 977) = 37.90, *p* < .001
Euphemistic Labelling	0.06	*F*(6, 987) = 11.70, *p* < .001	0.23	0.18	*F*(10, 977) = 22.80, *p* < .001
Advantageous Comparisons	0.13	*F*(6, 987) = 24.60, *p* < .001	0.37	0.25	*F*(10, 977) = 38.30, *p* < .001
Displacement of Responsibility	0.04	*F*(6, 987) = 8.42, *p* < .001	0.12	0.08	*F*(10, 977) = 9.99, *p* < .001
Diffusion of Responsibility	0.08	*F*(6, 987) = 15.10, *p* < .001	0.27	0.19	*F*(10, 977) = 26.30, *p* < .001
Distorting the Consequences	0.11	*F*(6, 987) = 21.50, *p* < .001	0.34	0.24	*F*(10, 977) = 35.40, *p* < .001
Dehumanization of Others	0.17	*F*(6, 987) = 37.50, *p* < .001	0.33	0.16	*F*(10, 977) = 23.50, *p* < .001
Attribution of Blame	0.13	*F*(6, 987) = 26.20, *p* < .001	0.34	0.22	*F*(10, 977) = 32.70, *p* < .001

Note. Full regression coefficients are found in S1 Appendix.

We also wanted to explore the incremental criterion validity of *phronesis* network components over and above Moral Foundations for additional morally salient outcomes. To that end, Table 16 also included morally salient aspects of personality. Moral Foundations explained variance in morally salient aspects of personality, ranging from 5% (Narcissism) to 14% (Honesty/Humility). *Phronesis* network components explained variance in all of these outcomes over and above Moral Foundations, however, ranging from +9% (Machiavellianism) to +23% (Sadism).

Finally, we tested for the degree to which *phronesis* network components would predict the propensity to morally disengage over and above Moral Foundations (see Table 16). Moral Foundations explained large amounts of variance in all outcomes of interest, ranging from 6% (Euphemistic Labelling) to 17% (Dehumanization of Others). As before, *phronesis* network components explained large amounts of variance in these outcomes of interest over and above Moral Foundations for all outcomes, ranging from +12% (Displacement of Responsibility) to +34% (Attribution of Blame).

### Study 3 Discussion

This study aimed to evaluate the criterion validity of our newly developed SPM, focusing on its ability to explain variance in various outcomes related to flourishing, personality traits, and moral disengagement. The findings provide strong support for the criterion validity of the *phronesis* measure, particularly in its prediction of flourishing across multiple dimensions. This is a crucial property of the new measure; while it is impossible for any measure to have complete construct coverage based on the contents of its items alone, criterion validity shows that we have captured the essence of the construct under study. That is, on aggregate, this measure functions as a measure of *phronesis* should, robustly predicting moral and flourishing variables.

#### *Phronesis* and flourishing

Our results indicate that the *phronesis* components collectively predict flourishing outcomes, with particularly strong associations found between *phronesis* and Character Strengths, as well as Meaning and Purpose. These findings are consistent with the theoretical grounding of *phronesis* in Aristotelian ethics, which posits that practical wisdom is central to leading a flourishing life. The weaker, yet still significant, relationship with Social Connectedness further supports the potential role of *phronesis* in facilitating positive social relationships, albeit to a lesser extent than its influence on individual moral strengths.

Interestingly, the perceptual components of *phronesis*, including Virtue Identification and Situational Moral Relevance and Irrelevance, did not show significant correlations with flourishing outcomes. This suggests that while these perceptual abilities may be important aspects of moral judgment, they are not associated with broader well-being outcomes measured in this study. The peripheral role of these perceptual components within the *phronesis* network, especially in the US sample, further highlights their distinct function compared to other, more central components like Moral Deliberation and Moral Self-relevance. This is consistent with the idea that knowing the good does not necessarily entail doing the good, and therefore flourishing is not necessarily downstream of moral perception. Additionally, we showed that *phronesis* is more than a mere correlate of flourishing, but also robustly predicts flourishing two months later.

#### *Phronesis*, personality, and moral disengagement

The *phronesis* components were also analyzed in relation to personality traits and the propensity to morally disengage. The positive correlations between Moral Deliberation, Moral Self-relevance, and Conscientiousness underscore how these aspects of *phronesis* align with a conscientious and thoughtful approach to moral decision-making. Notably, the distinction between Negative Moral Emotion and general trait Negative Emotion suggests that these constructs capture different aspects of emotional experience. Negative Moral Emotion is associated with a greater tendency to morally disengage, while also being closely related to Moral Self Relevance in the *phronesis* network, showing that practically wise people are emotionally invested in being (morally) good people. Additionally, the inverse relationships between *phronesis* components and Dark Tetrad traits, especially Moral Self Relevance, and the clear pattern of negative associations between *phronesis* components and moral disengagement, emphasize the potential of *phronesis* to mitigate tendencies towards morally disengaged behavior. However, the positive correlation between Aspired Moral Identity and Narcissism warrants further investigation, as it suggests that for some individuals, the pursuit of moral aspirations may be driven by self-interest or an inflated sense of self-importance.

#### Moral foundations

The relationships between *phronesis* components and Moral Foundations reveal important insights into how different aspects of moral reasoning and emotion interact. As expected, Emotional Regulation was negatively related to Moral Foundations in some cases, which aligns with the idea that Moral Foundations Theory emphasizes the role of basic emotions in moral judgment. This suggests that those who are adept at regulating their emotions may be less influenced by these foundational moral intuitions, potentially reflecting a more rational, rather than emotional, approach to morality. Similarly, Situational Moral Irrelevance was also negatively related to Moral Foundations on the whole, indicating that individuals who excel at identifying when a situation does not require moralization are less likely to rely on sentimentalist moral intuitions. This suggests that emotional moral thinkers might struggle to discern when a situation does not necessitate a moral response, possibly leading to over-moralization of situations.

On the other hand, components of *phronesis* that are engaged when a situation is deemed morally salient, such as Aspired Moral Identity, Moral Integration, and Moral Deliberation, were positively associated with Moral Foundations. This is perhaps unsurprising, as these aspects of *phronesis* are likely to be activated in scenarios where moral considerations are at the forefront, and sentimentalist thinkers might assume most situations are morally salient. However, the weaker performance in Situational Moral Irrelevance and Emotional Regulation among these individuals suggests that they might be prone to moralize situations unnecessarily and struggle with managing their emotional responses in morally charged contexts.

Given the mixed associations between *phronesis* network components and Moral Foundations, we sought to determine which set of constructs better predicts our key criterion variables in the flourishing network. The findings from our regression analyses revealed that while Moral Foundations explained a significant portion of the variance in flourishing outcomes, *phronesis* network components added substantial predictive power across nearly all outcomes, particularly in areas like Character Strengths and Meaning and Purpose. This suggests that while Moral Foundations provide a useful framework for understanding some aspects of flourishing, the *phronesis* network offers additional, and often superior, explanatory value. Moreover, when predicting morally salient aspects of personality, *phronesis* network components consistently explained more variance than Moral Foundations, particularly in traits associated with the Dark Tetrad. This highlights the robustness of *phronesis* as a construct that not only complements but also extends beyond the explanatory power of Moral Foundations. Finally, in terms of the propensity to morally disengage, *phronesis* network components again outperformed Moral Foundations in predicting this critical outcome. The ability of *phronesis* components to account for substantial additional variance in moral disengagement indices underscores their relevance and utility in understanding moral behavior, especially given that moral disengagement is a more proximal moral outcome compared to flourishing. In this way, *phronesis* has been shown here to predict both proximal (morality) and distal factors (flourishing) over and above Moral Foundations.

## General discussion

The findings presented in this article represent a turning point for the study of *phronesis*, combining rigorous empirical methods with ambitious theoretical innovation. Our work offers a clear step forward in the long-standing dialogue between philosophy and psychology, challenging theoretical constructs that have held sway for over 2,300 years while providing practical tools to drive a new empirical research agenda. In doing so, we have achieved three critical goals: the production of a validated, fairly short, and accessible measure of *phronesis*; the reconceptualization of Aristotelian theory (i.e., the four-component APM was amended to become the neo-APM network) through empirical data; and the application of cutting-edge psychometric techniques to offer novel insights into the structure and functioning of practical wisdom.

### The viability of the construct, and the challenge to theory

Grounding a psychological construct and instrument in a philosophical model such as the four-componential APM—and an ancient one at that—invites a welter of controversy. For present purposes, it suffices to note that in contrast to earlier empirical work on *phronesis* [19], which commenced with a CFA aimed explicitly at confirming the essential structure of the APM, we decided to follow a bottom-up inductive approach, beginning with an EFA, and not to take any previously theorized structure as given. Studies 1a–1b established (through EFA and CFA) the potential viability of a ten-factor structure in representative UK and US samples, rather than four factors implied by the APM. Moreover, bifactor models could not establish that the ten neo-APM factors could be accommodated under an overarching APM framework.

The relevance of the non-confirmation of a four-factor structure—which had been previously confirmed in much smaller and non-representative samples [19]—for the viability of an Aristotelian virtue ethical understanding of *phronesis* should not be understated, but it must not be overstated either. At the most general level, while analyses by psychologists and philosophers of the same psycho-moral constructs may produce synergic value-addedness at the best of times [75], such crossover work is not a marriage made in heaven [86]. Virtue ethicists among philosophers and virtue theorists among psychologists may well share an interest in making sense of Aristotle’s claim for *phronesis* as excellence in moral decision-making and its association with the good, flourishing life, but beyond that point their methodologies and interests will diverge. Once philosophers have distilled the four aims or functions of *phronesis* from Aristotle’s original texts and created an Aristotelian *phronesis* model (APM) with named components, psychologists will want to test whether *phronesis* really manifests itself via four discrete factors. From a psychological point of view, it may appear as a shock to the system for philosophers that this does not seem to be the case.

However, a philosopher might use the analogy that even if a company is established with four functions in mind and four departments are created to execute these functions, there is no particular reason to think that a subsequent analysis of how these functions are taken care of within the company reflect the departmental barriers. Some functions originally intended for Department X may in fact be partly performed in Department Y, etc. This does not matter as long as the overall *aims* of the company are fulfilled collectively. Notice that the concept of “function” in the context of understanding *phronesis* might be better understood as referring to what *phronesis* ultimately aims to achieve or predict, rather than how it is structured in the decision-making process. In other words, the four Aristotelian “functions” of *phronesis* could be seen as the broader categories of outcomes to be predicted, or the purposes that *phronesis* serves—what it functions to accomplish in terms of moral and practical outcomes. The ten factors, on the other hand, represent the specific components or processes involved in *phronesis* as it operates in decision-making that might be intervened upon. “Function”, in the philosophers’ lexicon, might thus be read as “purpose,” not “process.” Therefore, the APM functions in Table 17 may still be tenable as such, but the APM processes are much more nuanced than previously hypothesized.

**Table 17:** Comparing the APM and the neo-APM.

Hypothesized APM process	Theorized APM function/purpose	Empirically derived SPM components	Centrality for neo-APM	Temporality for neo-APM (before or after action choice)
Moral Perception	Constitutive Function	Virtue Identification	Peripheral	Before
		Situational Moral Relevance	Peripheral	Before
		Situational Moral Irrelevance	Peripheral	Before
Moral Identity	Blueprint Function	Moral Self Relevance	Central	Before
		Aspired Moral Identity	Central	Before
Moral Emotion	Emotional Regulative Function	Emotional Regulation	Peripheral	Before
		Positive Moral Emotion	Central	After
		Negative Moral Emotion	Central	After
Moral Adjudication	Adjudicative Function	Moral Deliberation	Central	Before
		Moral Integration	Central	Before

Previous models of *phronetic* decision-making did not typically consider any particular variables to be more central than others to the moral decision-making process. One of our key findings was that some of the ten *phronesis* sub-components appeared to be more central to the *phronesis* network compared to the rest. Those were variables to do with adjudication, including Moral Deliberation and Moral Integration, variables related to moral identity, namely Moral Self Relevance and Aspired Moral Identity, and Moral Emotion, especially Positive and Negative Moral Emotion. Yet we must think critically about what this means. As we included cross-sectional data only within the network analyses, we cannot infer causal relationships between variables from these findings. However, we might reasonably expect that Moral Deliberation and Moral Integration *follow* other variables in the network and could be *outcomes* of, say, first considering being a “good” person to be personally relevant (i.e., Moral Self Relevance) given the emotional impact of having done good (Positive Moral Emotion) or bad (Negative Moral Emotion), and therefore aspiring to improve oneself morally (i.e., Aspired Moral Identity). Therefore, given the centrality of moral emotion- and identity-related variables, and given that they likely precede the other central aspects of the network, this would appear instructive for educators. Specifically, we believe our data suggest that establishing Moral Self Relevance through an exploration of the emotional effects of morally salient scenarios, and then Aspired Moral Identity, may be the most effective first steps for evidence-based moral/character education ultimately aimed at *phronesis* development as these are most likely to affect other aspects of the *phronesis* network, and indeed, flourishing.

Within the preamble for Study 2, we referred to the conceptualization of a latent *Phronesis* factor as potentially devolving into an infinite regress where we are tasked with explaining what, in turn, causes *phronesis*. Using network analyses, we have gone beyond this conceptualization to suggest that if we grant the likelihood of adjudicative factors (Moral Deliberation and Integration) being downstream of the identity factors (Moral Self Relevance and Aspired Moral Identity), with the central emotional factors (Positive and Negative Moral Emotion) downstream of adjudication (per the question phrasing in the SPM), then the identity and emotion factors might constitute organizing factors within the network overall as intrinsic motivators. Conceptualized as a network with emotion and (especially) moral-identity-related factors at the center, *phronesis* constitutes a bedrock organizing principle, rather than *phronesis* operating as a sort of “God of the gaps” in wisdom research, potentially rendering the concept redundant [37, 39]. Krettenauer [50] may be right that, from a structural point of view as well as the point of conceptual parsimony, some of the functions originally identified in the APM (moral perception variables and emotion regulation) might better be seen as preconditions rather than constituents of *phronesis*, which would then leave three essential functions only: moral identity, informed by moral emotion, and moral adjudication potentially downstream of this. This suggestion would seem to align with our data, as the network analysis in Study 2 found that the nodes acting as the most crucial information conduits within the *phronesis* network relate to moral identity and adjudication functions, with Positive and Negative Moral Emotion still being important but as “preconditions” for Moral Self Relevance.

**Fig 5:**
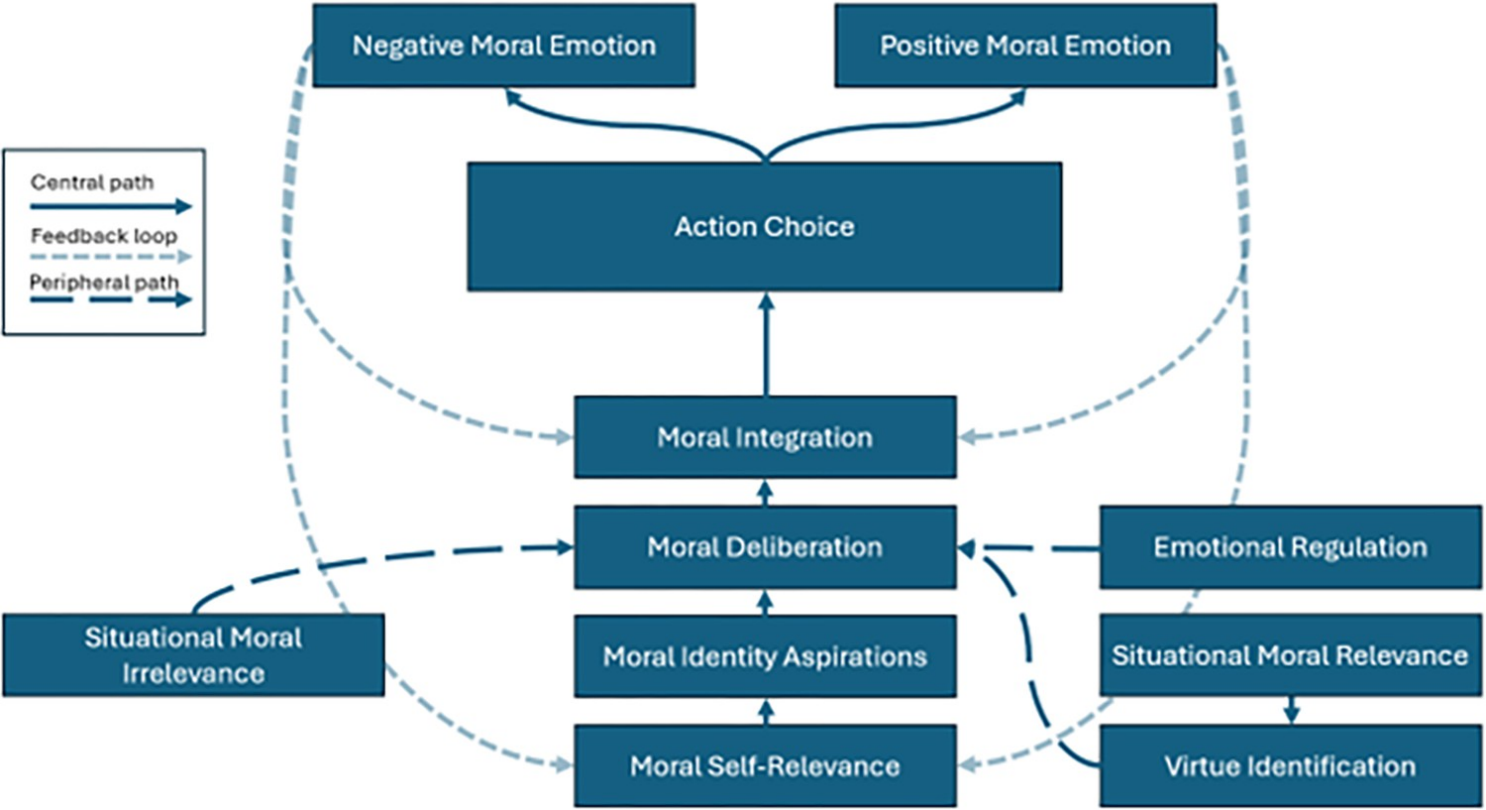
Phronetic decision-making per the neo-APM.

While future research may seek to further disentangle the temporal dynamics of the SPM components, the data on the relative centrality of these components points to a “central path” in *phronetic* decision making, with other variables in the network being more peripheral. Informed by this data, Fig 5 presents the neo-APM as a simplified network model of *phronesis*, for practical purposes.

### Future research directions

How might the findings in this article shape future research agendas regarding *phronesis* and, more generally, human flourishing as partly constituted by *phronesis*? In order to answer this question briefly here at the end, we need to revisit some alternative conceptions of *phronesis* mentioned in the Introduction and explore how those would fare in light of our findings. According to the Aretai (Center) Model, all ethical virtues are ultimately unified within *phronesis* itself, understood as overall *moral expertise* [40]. According to the APM and the neo-APM, in contrast, discrete individual moral (and civic) virtues provide the initial motivation for virtuous actions in children; and they continue to drive virtuous action even after *phronesis* has (if all goes well) latched itself onto the individual habituated virtues, in adolescence and early adulthood. So, for example, when the virtuous agent experiences an apparent conflict between honesty and compassion, it is the primary motivation stemming from those discrete virtues that drives her ultimate action, although the secondary motivation from *phronesis*—prompting the search for an integration or some sort of adjudication—facilitates the decision-making process.

Some of the data from our present studies help us to adjudicate between the conceptualizations on offer in the APM, neo-APM, and Aretai Model. Unlike the neo-APM, the APM does not speak to the relative centrality of some components compared to others. On the other hand, the Aretai Model conceptualizes *phronesis* as “expertise”, and the central components of the neo-APM are difficult to conceptualize that way (e.g., Aspired Moral Identity). We agree with Han that the Aretai Model and APM may be essentially compatible and simply be looking at the same construct from different angles [42], at least if we explore it cross-sectionally. One important difference between the APM/neo-APM models and the Aretai Model is developmental (and, by implication, educational) and can only be arbitrated through longitudinal studies of *phronesis* development and cultivation: Do the individual virtues develop separately first and not come together until children begin to realize that the same situation can call for conflicting virtues, perhaps not until middle childhood [87], or does ethical expertise develop as a unitary capacity and then later diversify into different virtue streams? We hope that this empirical and conceptual discourse will continue to evolve in future research.

Another sort of research that might offer a clue comes from neuroscience. As Han [43] notes, the fact that fMRI experiments demonstrate significant activity in, and interactions between, different regions in the brain (involving multiple circuitries) during moral tasks does not bode well for the subsumption idea that underlies the Aretai Model. We would like to add that the centrality of the moral-identity factors in the network analysis in Study 2 does indicate the presence of a highly cognitive and conscious core activity in *phronesis*, which seems to speak against the model of *phronesis* as general expertise; that is, if we understand expertise as essentially involving tacit knowledge and an experientially learned *skill*, as seems to be the standard understanding of “expertise” in moral psychology [88]. All in all, however, future comparisons between the neo-APM and Aretai Models need to await further evidence from developmental science.

Posing a challenge to both the APM (and neo-APM) and the Aretai Model is the specter of eliminativism: the idea that *phronesis* may be a psychologically and theoretically redundant construct [37, 39]. The eliminativist approach posits that rather than appealing to one (multi-componential) intellectual virtue that is supposed to carry out the functions neo-Aristotelians ascribe to *phronesis*, we should appeal to a distinct trait performing each function. There are two logical options here: (a) all the functions of *phronesis* can be reduced to those of the individual moral virtues, or (b) there are discrete psychological processes (such as metacognition and moral identity) performing each function independently on behalf, so to speak, of one or more virtues. Lapsley [37] argues for (b) and it seems to be Miller’s [39] favored option also. In either case, “practical wisdom does not exist”.

If (a) were true, one would expect to see radically different procedures at work in decision-making processes depending on which virtues feature in the problematic quandaries at hand. However, the study by Feraco et al. [41] speaks strongly against (a), where specific virtue factors did not perform well as predictors over and above a general factor. Option (b) seems to be the more plausible one. Yet we have argued elsewhere that (b) betrays an inadequate understanding of the network-and-coordination nature of our moral system—akin to the misunderstanding that the nature and cultivation of decathlon as a sport can be reduced to the ten individual sports that comprise it [89]. In a similar vein, Han’s [42, 43] overviews of network analyses of moral functioning and of neuroscientific evidence about moral decision-making reveal that inter-constituent cooperation and coordination between different functional realms is required for a reasonable moral decision to ensue. More generally speaking, according to network theory, a network with connecting nodes can generate significantly more information than the sum of the nodes without connectivity [90]. The studies in the present article indicate that thinking of *phronesis* as a network rather than a composite entity, as in Darnell et al. [19], may be more informative. Indeed, we would argue that if *phronesis* is conceptualized as a network of interrelated variables, in which some variables are more central than others (as explained above), the redundancy objection no longer applies. Future research may help to shed further light on this debate.

Finally, in this section on future research directions, some observations are in order about the relevance of our findings for future research on human flourishing. The main problem from a neo-Aristotelian perspective about our previous empirical and psychometric work [19, 51] was that it failed to measure the relationship between *phronesis* on flourishing (*eudaimonia*), which would be a crucial test for the criterion validity of the APM (or revised neo-APM). Given Aristotle’s foregrounding of flourishing, we chose a comprehensive wellbeing scale [59] derived from a flourishing paradigm [46]. Once we had established, in Studies 1a–1c, a ten-componential model of *phronesis*, the next step was to explore how it correlates with flourishing. Indeed, perhaps the most significant single finding of the current article—at least in the sense of being propitious with regard to answering the question about the credibility of the neo-APM—is the one recorded in Study 3 about how well the confirmed *phronesis* factors correlate with flourishing and its proposed sub-factors.

The ones that correlate least with flourishing are the components that would fall theoretically under moral perception in the APM. That is not altogether surprising. Already in Darnell et al. [19], moral perception had demonstrated the lowest standardized factor loadings in relation to composite *phronesis*. There are sound developmental reasons to suggest that moral perception is a crucial factor in the early years of *phronesis* development, when learners are figuring out what really matters and how the world is constituted morally, but that it may wane in importance once they grow older and become more experienced with the ups and downs of adult life in general (unless they enter a new professional field where novel perceptive insights are called for [91]). The focus then moves from simply understanding and interpreting what is going on to actually relating it to one’s blueprint of the good life and subjecting it to deliberation and adjudication. Notably, all our respondents this time were adults.

The finding about a link between *phronesis* and flourishing chimes in well with psychological research on general wisdom and wellbeing. While we could not identify studies using the same wellbeing scale as we did, objective wellbeing has typically been measured in wisdom studies using Ryff’s [92] measure of “psychological wellbeing,” which is generally considered to prioritize flourishing over subjective wellbeing. The overall finding from the literature is that wisdom is moderately-to-largely correlated with flourishing but less so with subjective wellbeing—and in the short term may even diminish subjective wellbeing as wise decisions are often painful [93–95]. The SPM may offer an opportunity for more research into understanding post-*phronetic* pain, and other such barriers to practical wisdom. In a recent paper, Glück and Westrate [17] advise wisdom researchers to look beyond a linear relationship. They hypothesize and partly confirm that the relationship between wellbeing and wisdom is triangular rather than linear, with highly wise people typically being high in wellbeing but people high in wellbeing (even flourishing) not necessarily being highly wise. Future research into the dynamics of the (practical) wisdom and flourishing relationship may be able to shed light on where these two constructs diverge.

Shortly before submitting the present article, a new paper appeared in print that purports to do the same as we have aimed for here: namely, to empirically test Aristotle’s link between practical wisdom and flourishing [96]. Based on survey data from 230 US undergraduates, the authors established that wisdom at the beginning of the semester significantly predicted flourishing at the end of the semester. While we applaud the intentions behind this study and deem it obliquely to confirm our thesis (the small and non-representative sample size notwithstanding), we cannot avoid expressing a couple of theoretical misgivings. First, the wisdom measured by the authors is not practical wisdom in Aristotle’s sense but general wisdom as tested through Ardelt’s [97] three-dimensional wisdom scale. Second, Ardelt and Kingsbury [96] measure flourishing, drawing on Ryff [92], via four indicators of flourishing: self-acceptance, mastery, purpose in life, and growth orientation. Three of those four indicators only scratch the surface of Aristotle’s flourishing theory, and the fourth (self-acceptance) seems alien to it. In contrast, our measure draws upon Aristotle’s own conception of *phronesis*, and although no existing measure exists that aims to capture fully Aristotle’s theory of *eudaimonia*, we chose the one that comes closest to acting as a proxy for it (see 47,60): for instance, by including “character and virtues” explicitly as one of the components [76]. No doubt, Ardelt and Kingsbury [96] have successfully tested a theory of a relationship between wisdom and flourishing, but it is not literally speaking a test of *phronesis* and *eudaimonia*, as Aristotle and neo-Aristotelians understand these constructs, whereas ours is. Hence, the current findings constitute the first empirical confirmation of a link between *phronesis* and flourishing, which will have significant implications for future research directions in both character education and within the applied wellbeing professions.

### Limitations

As with any study, there are limitations to consider. We gathered online samples via Prolific Academic, so there is some possibility of selection bias if a particular kind of person does or does not tend to use it compared to others. For example, highly paid professionals may not seek to supplement their incomes in the same proportions by doing online surveys. Nonetheless, this study’s samples are at least nationally representative of the UK and US based on their distribution of age, sex, and ethnicity, and our analyses are well-powered, allowing us to make general claims about *phronesis* within the national populations in question. We should caution, however, that the findings reported here may or may not hold up in sub-sections of the population that think/behave differently from the general population. Nonetheless, establishing what is generally the case is an important first step for a research program such as this. Future research should therefore seek to understand how *phronesis* operates within particular sub-samples of the population, such as within particular professions. Further alleviating concerns about the online data collection platform is evidence suggesting low levels of dishonesty, as well as high levels of participant comprehension [53]. These concerns are mitigated further by “attention checks” embedded within our surveys to help screen out poor quality responses, as well as our own checks that participant demographics align with published census data.

Another limitation is that this study involved two Western populations: the UK and the US. We do not view this as a problem *per se* since *phronesis* is borne out of the Western philosophical canon. Indeed, this would seem the obvious place to start. However, future research exploring *phronesis* in more diverse cultural and sociobiological contexts would likely shed further light on the ubiquity of the principles identified here. For instance, in some cultures (e.g., authoritarian ones), it may not be the case that *phronesis* predicts flourishing, especially if other factors are valued more within those cultures. Likewise, the factor structure of *phronesis* could look different outside of the US or UK. It is our hope that researchers will be able to use the SPM to shed light on these kinds of issues within the coming years.

In this study, we aimed to develop a measure of *phronesis* based on an initial item set that covered more conceptual contours than its predecessor. At the same time, the only previous measure of *phronesis* took 45+ minutes to complete, and so we wanted to develop a measure that could be completed comparatively quickly to ensure its practical utility. Moreover, publications introducing the previous *phronesis* measure [19, 76] did not specify all the scoring instructions. To build on these limitations, we needed to strike a balance between having a usable and wieldy measure and having a measure that could adequately assess this complex construct. This was a difficult task, and we undoubtedly did not include every variation of possible items in the initial item pool. However, these concerns are mitigated by the demonstrated criterion validity of the overall measure, in that it correlates with various outcome measures in theoretically congruent ways. We believe the outcomes assessed here were fit for purpose and allowed us to test Aristotle’s core hypothesis that practical wisdom predicts key indices of flourishing. *Phronesis* was also associated with a range of other variables of interest. However, there are other aspects of human flourishing that may be associated with *phronesis* that future research could uncover, such as academic outcomes, relationship satisfaction, or professional success.

These findings are likely to be of interest to those engaging in character educational practice. However, as this paper involved all-adult samples, practitioners should exercise caution in generalizing these findings to younger populations, where we expect that factors such as Virtue Identification, Situational Moral Relevance, and Situational Moral Irrelevance may be more important than shown here. Our findings may have more direct relevance to adult character education (e.g., within the professions or universities). That said, the lack of a comprehensive *phronesis* measure for younger age groups makes the current investigation the most informative empirical account of *phronesis* for character education with young people to date, especially when considering the longer-term outcomes of school-based character education.

This study included a two-month longitudinal component, moving beyond cross-sectional data for purposes of establishing test-retest reliability and predictive validity. However, this is a limited longitudinal timeframe. To further establish the predictive power of *phronesis*, and perhaps more importantly to establish its causal role, more longitudinal studies are needed (e.g., experiments, cohort studies, etc.).

#### Concluding remarks

The empirical studies undertaken and described in this article have provided a revised model of Aristotelian *phronesis* (the neo-APM)—originally theoretically motivated only—and yielded a practicable instrument to measure *phronesis*. Our studies have demonstrated meaningful correlations between *phronesis* and flourishing and have unearthed various other associations that either confirm previous studies of wisdom in psychology or add to the knowledge basis about the narrower construct of practical wisdom. The data set we gathered is large and some of it still awaits further analysis in subsequent papers. More research in this area is urgently called for, for example research comparing the neo-APM more directly with wisdom as understood in psychology (see 87), although that task is complicated by the fact that there are so many wisdom conceptions available [98], and the internal correlation between them rarely exceeds .3 [99].

We choose to end this discussion by returning to the most “controversial” premise of this article, noted earlier. Why conduct a series of psychological studies to demonstrate the credibility of a construct that is of pure philosophical provenance, and over 2,300 years old at that? This question hides a deeper one: what does it mean to conduct an empirical analysis based on a specific explicit realist moral ontology, such as neo-Aristotelian virtue ethics? This endeavor can be interpreted in many ways, ranging from a weak reading to a strong reading of the extent to which the ontology in question informs the research orientation.

We have rejected the strong reading that a philosophical ontology should *constrain* the research process, by deciding to follow a bottom-up approach, rather than beginning with CFA to consolidate a pre-existing model as in our previous work [19]. We thus wish to avoid the critique that our approach is revanchist and involves special pleading to a long-gone philosopher [37]. Nevertheless, we acknowledge that how we gathered and interpreted our data has unavoidably been affected by our motivation to establish the credibility and serviceability of the Aristotelian model that we confirmed, in a revised incarnation, in Studies 1a–1c.

We remain unrepentant about our ontological assumptions because we believe no social scientists can avoid them. All research in moral psychology presupposes some implicit moral ontology that steers the analysis, and our uniqueness lies simply in making this ontology explicit rather than implicit. For example, Kohlberg’s [1, 20] enterprise was motivated by his antipathy toward moral relativism, and he gathered evidence that he hoped would rebut such an ontology (and which it did). *Phronesis* (in a neo-Aristotelian sense) does not become anyone’s chosen area of study unless they are committed to a realist moral ontology, which assumes that there are better or worse answers to moral quandaries, and that there is an essentially intellectual process, which one can cultivate through moral learning, that helps one deliberate and decide upon the better answers and stay away from the worse ones.

All in all, this article has provided evidence of a method through which a philosophical construct can mature into a fully-fledged psychological one. More generally, it has confirmed our assumption about the potential fruitfulness of crossover work between philosophy and psychology. However, such work requires a bridging effort from both sides—rather than philosophical ideas being removed from empirical scrutiny, or psychology ignoring ancient (or even modern) wisdom from adjacent fields. This article has not only demonstrated the viability of transforming a philosophical construct into a psychological one but also highlighted the importance of cross-disciplinary collaboration. More specifically, the neo-APM, as presented in this article, represents a significant stride in the empirical measurement of *phronesis*. It encapsulates a comprehensive, multi-dimensional approach that integrates theoretical depth with empirical rigor. The measure based on this model is not merely an academic tool; it is a gateway to understanding key dynamics of moral virtue and behavior across various life stages and societal challenges, marking an important contribution to the field of moral psychology. The neo-APM and its associated measurement tool (the SPM) thus offer new avenues for research and application, with the potential to reshape our understanding of moral psychology and its practical implications. We believe that this work marks a significant step forward, not just for the study of *phronesis* but for the broader field of psychology, which stands to benefit greatly from continued integration with philosophical insights.

## Supporting information

S1 Appendix (DOCX)
