# Supplementary material for: Was Aristotle right about moral decision-making? Building a new empirical model of practical wisdom
Source: PLoS One. 2025 Jan 22;20(1):e0317842. doi: 10.1371/journal.pone.0317842 (PMC11753716; doi:10.1371/journal.pone.0317842)
Supplement: S1 Appendix — (DOCX) [file pone.0317842.s001.docx]

Appendices

**Appendix A: Short Phronesis Measure**

**About**

This measure contains ten sub-factors, broadly fitting with a hierarchical four-component model:

**Moral Adjudication**

1. Moral Deliberation
   (tendency to seek necessary information before making a morally salient decision)
2. Moral Integration
   (tendency to make moral decisions by integrating many salient factors)

**Moral Identity**

1. Aspired Moral Identity
   (tendency to strive to become one’s best moral self)
2. Moral Self-relevance
   (belief that one should aspire to become good)

**Moral Perception**

1. Virtue Identification
   (correctly labelling virtues relevant to a given scenario)
2. Situational Moral Relevance
   (correctly identifying that a situation requires a decision that affects one’s character)
3. Situational Moral Irrelevance
   (correctly identifying that a situation does not require a decision that affects one’s character)

**Moral Emotion**

1. Emotional Regulation
   (cross situational ability to regulate one’s emotions)
2. Positive Moral Emotion
   (experiencing positive emotion when one behaves morally)
3. Negative Moral Emotion
   (experiencing negative emotion when one behaves immorally)

These measures can be used on their own as discrete measures. It is not strictly necessary to use all sub-measures in every study if they are not relevant.

These measures can be used to create higher order factors (i.e., *Phronesis, Moral Adjudication, Moral Identity, Moral Perception,* or *Moral Emotion*), but as they are scored on different scales, they should be converted to Z-scores first.

IMPORTANT NOTE TO PRACTITIONERS: Researchers may decide to use total or average scores for each measure. To assess individuals for educational purposes, it is better to convert total scores for each measure to percentile ranks. This lets you know the percentage of people an individual scores higher than in the wider population. The key for converting raw scores to percentile ranks can be found at <https://tinyurl.com/spmdevelopment>.

Please contact the authors with any queries or if you wish to translate this measure. This measure is free to use for researchers, but please cite this journal article.

PLEASE CONTINUE TO THE NEXT PAGE FOR THE FULL MEASURE

**Moral Deliberation, Moral Integration, Aspired Moral Identity, and Moral Self-relevance**

**Researcher Instructions:**

This self-report questionnaire includes a range of standard self-report items, with different item combinations representing different components of *Phronesis*. When validating this measure, items below were presented in a random order (i.e., the statement order, but not the Likert scale anchors). However, for presentation purposes here, they are grouped together by construct. To avoid potential confounds, please endeavour to present the items below in a random order wherever possible. This should be possible within most online survey software. Mixing the items up quasi-randomly may suffice for paper surveys.

While it is possible to include items from one sub-measure and not others if it makes sense for your research, this may introduce some biased responding in your data, so please bear this possibility in mind.

**Participant Instructions:**

The next section asks you about how you generally think and behave.

Please indicate the degree to which you agree with the statements below as honestly as you can.

|  |  | **Strongly disagree** | **Somewhat disagree** | **Neither agree nor disagree** | **Somewhat agree** | **Strongly agree** |
| --- | --- | --- | --- | --- | --- | --- |
| 1 | I make sure to gather all the details before forming an opinion about what’s right and wrong. |  |  |  |  |  |
| 2 | In difficult situations, I try to look at things from different angles. |  |  |  |  |  |
| 3 | I think it’s important to know the full story before making a judgement. |  |  |  |  |  |
| 4 | I believe that understanding the situation fully is crucial to making a good decision. |  |  |  |  |  |
| 5 | Understanding all sides of a story helps me determine what’s right. |  |  |  |  |  |
| 6 | I try to dig deeper to understand the root of a problem. |  |  |  |  |  |
| 7 | Asking the right questions is key to making wise decisions. |  |  |  |  |  |
| 8 | Getting a full picture of a situation is crucial for me before deciding. |  |  |  |  |  |
| 9 | I never make a decision without exploring all the facts. |  |  |  |  |  |
| 10 | I make sure to get all the perspectives before deciding on a course of action. |  |  |  |  |  |
| 11 | I don’t rush decisions. I take time to understand the situation. |  |  |  |  |  |
| 12 | I seek to understand all aspects before deciding what’s right or wrong. |  |  |  |  |  |
| 13 | If I don’t know enough about a situation, I’ll take the time to learn more. |  |  |  |  |  |
| 14 | I make conscious efforts to recognize and minimize my own biases before making a decision. |  |  |  |  |  |
| 15 | I evaluate the reliability and credibility of the information sources before making a judgment. |  |  |  |  |  |
| 16 | I look for trustworthy sources when gathering information for a decision. |  |  |  |  |  |
| 17 | I regularly check the validity of my information sources. |  |  |  |  |  |
| 18 | I tend to make decisions about right and wrong after considering all the available information and emotions. |  |  |  |  |  |
| 19 | In deciding right from wrong, I consider all my thoughts, feelings, and information. |  |  |  |  |  |
| 20 | It’s important to me that I live up to the expectations I set for myself. |  |  |  |  |  |
| 21 | When I face difficult decisions, I consider what aligns best with my principles. |  |  |  |  |  |
| 22 | When faced with challenging situations I ask myself what a good person would do |  |  |  |  |  |
| 23 | I believe personal growth involves improving my understanding of what is right and wrong. |  |  |  |  |  |
| 24 | I strive to become a person that others can look up to. |  |  |  |  |  |
| 25 | I set personal targets that involve improving my ethical awareness and character. |  |  |  |  |  |
| 26 | I aim to make decisions that don’t just benefit me in the present, but also align with my long-term ideals. |  |  |  |  |  |
| 27 | I try to consider how my decisions today will reflect on the person I aspire to be in the future. |  |  |  |  |  |
| 28 | I often reflect on the progress I have made in aligning my actions with my ideals. |  |  |  |  |  |
| 29 | I believe my actions should reflect the type of person I aspire to be. |  |  |  |  |  |
| 30 | I question how my decisions align with societal expectations and values. |  |  |  |  |  |
| 31 | What I believe about myself influences my decisions. |  |  |  |  |  |
| 32 | The kind of person I want to be shapes my actions. |  |  |  |  |  |
| 33 | The type of person I aim to be influences my decisions. |  |  |  |  |  |
| 34 | The person I want to be influences how I judge right from wrong. |  |  |  |  |  |
| 35 | I strive to act in a way that is consistent with my personal beliefs. |  |  |  |  |  |
| 36 | I feel uncomfortable when my actions do not match my values. |  |  |  |  |  |
| 37 | Maintaining my integrity is crucial to my self-worth. |  |  |  |  |  |
| 38 | Consistency between my actions and my values enhances my self-respect. |  |  |  |  |  |
| 39 | Whether or not my actions harm others doesn’t affect how I see myself. |  |  |  |  |  |
| 40 | My integrity doesn’t play a big role in my self-worth. |  |  |  |  |  |
| 41 | My feelings can influence my view of what’s right and wrong. |  |  |  |  |  |
| 42 | The way I handle a situation can change depending on the circumstances. |  |  |  |  |  |
| 43 | The way I think, feel, and judge is closely connected. |  |  |  |  |  |
| 44 | My decisions are influenced by a mix of my thoughts, feelings, and personal beliefs. |  |  |  |  |  |
| 45 | I use my feelings, beliefs, and understanding differently depending on the situation. |  |  |  |  |  |
| 46 | My understanding, emotions, and values can change the way I make a decision. |  |  |  |  |  |
| 47 | My decisions are a mixture of my understanding, feelings, and values. |  |  |  |  |  |
| 48 | The situation often influences how I think, feel, and act. |  |  |  |  |  |

**Scoring:**

Reverse score items 39 and 40.

Items 1-19 = Moral deliberation

Items 20-34 = Aspired Moral Identity

Items 35-40 = Moral Self-relevance

Items 41-48 = Moral Integration

**Emotional Regulation**

**Researcher Instructions:**

Once again, these items should be randomised wherever possible**.**

**Participant Instructions:**

Below, you will find several scenarios that might provoke an emotional response.

For each situation, take a moment to reflect and imagine how you would typically react and then rate your ability to manage your emotions in that situation.

By ‘manage’, we mean your ability to keep your feelings from overwhelming you and to maintain your composure.

Please provide your answers on the following scale:

1 = Very poor ability to manage emotions

2 = Poor ability to manage emotions

3 = Neither poor nor good ability to manage emotions

4 = Good ability to manage emotions

5 = Very good ability to manage emotions

Please answer as honestly as you can.

|  | Very poor | Poor | Neutral | Good | Very good |
| --- | --- | --- | --- | --- | --- |
| You accidentally spill a drink on your clothes just as you are about to leave the house. |  |  |  |  |  |
| You are waiting for a bus and it’s running late, making you late for an appointment. |  |  |  |  |  |
| A friend cancels your plans together at the last minute. |  |  |  |  |  |
| You prepare a meal for others, but they don’t seem to appreciate it. |  |  |  |  |  |
| You find out that someone has been spreading untrue rumours about you. |  |  |  |  |  |
| You’re in a queue at the shop and someone pushes in front of you. |  |  |  |  |  |
| Your neighbour plays loud music late at night, disturbing your peace. |  |  |  |  |  |
| You make a small mistake and someone criticises you harshly. |  |  |  |  |  |
| Someone close to you doesn’t follow through on a promise they made to you. |  |  |  |  |  |
| Someone else gets recognition for something that you have achieved. |  |  |  |  |  |
| Your boss assigns you additional work just as you were leaving for the day. |  |  |  |  |  |
| You arrive at a restaurant for a reservation, but they’ve lost your booking. |  |  |  |  |  |
| Your co-worker makes a joke at your expense in a meeting. |  |  |  |  |  |
| Your flight gets cancelled and you’re left stranded in an unfamiliar city. |  |  |  |  |  |
| You get a parking ticket even though you’re sure you parked legally. |  |  |  |  |  |
| An online order arrives and the product is not what you expected. |  |  |  |  |  |
| Your computer crashes and you lose hours of unsaved work. |  |  |  |  |  |
| You’re stuck in traffic when you’re already running late. |  |  |  |  |  |
| A stranger is rude to you in a public place for no apparent reason. |  |  |  |  |  |
| You’re in a rush and the person in front of you is walking incredibly slowly. |  |  |  |  |  |

No items to reverse score. All measure Emotional Regulation.

**Virtue Identification**

**Researcher instructions:**

The statements below should be presented in a random order where possible. The order of virtues in the four columns to the right should also be randomised wherever possible.

Note that, in this version, correct answers are highlighted in bold for ease of communication within this document. However, they should not be highlighted in bold when presented to participants.

**Participant Instructions:**

In this section, you will be presented with different scenarios, and four character traits that may or may not be relevant to the situation.

Your job will be to select the **two that you feel are most relevant** to the situation.

|  |  | **Honesty** | **Loyalty** | Generosity | Moderation |
| --- | --- | --- | --- | --- | --- |
| **1** | **Your best friend reveals they cheated on an important exam and asks you to keep it a secret.**  You need to decide what to do in this situation. Which of the following are most relevant to your decision? |  |  |  |  |
|  |  | **Loyalty** | Generosity | **Honesty** | Patience |
| **2** | **You learn a loved one has committed a serious crime.**  You need to decide what to do in this situation. Which of the following are most relevant to your decision? |  |  |  |  |
|  |  | Diligence | Optimism | **Forgiveness** | **Assertiveness** |
| **3** | **You learn a coworker has been sharing false, damaging rumors about you.**  You need to decide what to do in this situation. Which of the following are most relevant to your decision? |  |  |  |  |
|  |  | **Honesty** | Kindness | Humor | **Ambition** |
| **4** | **You are given credit for a successful project you did not contribute to.**  You need to decide what to do in this situation. Which of the following are most relevant to your decision? |  |  |  |  |
|  |  | Diligence | **Loyalty** | **Honesty** | Ambition |
| **5** | **A friend asks you to lie for them in a situation that could get them in trouble.**  You need to decide what to do in this situation. Which of the following are most relevant to your decision? |  |  |  |  |
|  |  | Generosity | **Honesty** | Politeness | **Ambition** |
| **6** | **You have the opportunity to cheat on a test you are not prepared for.**  You need to decide what to do in this situation. Which of the following are most relevant to your decision? |  |  |  |  |
|  |  | **Integrity** | Kindness | **Loyalty** | Creativity |
| **7** | **Your boss asks you to do something unethical for the good of the company.**  You need to decide what to do in this situation. Which of the following are most relevant to your decision? |  |  |  |  |

**Scoring:**

Correct answers are highlighted in bold above; please change these to regular font before using. For each correct answer, award a score of 1. The maximum score for each question is therefore 2, and the minimum is 0.

**Situational Moral Relevance and Situational Moral Irrelevance**

**Researcher Instructions:**

The presentation order of the items below should be randomised where possible.

**Participant Instructions:**

In the following section, you’ll encounter a series of scenarios. Each one presents a situation that requires a decision.

Your task is to determine which scenarios involve decisions that could impact **your character**.

Reflect on these situations and consider the moral or ethical implications they might have on the individual involved.

|  |  | What I decide to do in this scenario  **does not**  affect my character | What I decide to do in this scenario  **does**  affect my character |
| --- | --- | --- | --- |
| 1 | Your best friend confesses they’ve been embezzling funds from their employer. They’ve asked you to keep this secret. |  |  |
| 2 | You’ve found a lost wallet full of cash. The owner’s identification is inside it. |  |  |
| 3 | You’ve discovered a colleague is taking credit for your work but confronting them might cause tension in your team. |  |  |
| 4 | You overhear a coworker making discriminatory comments, but reporting it might affect your working relationship. |  |  |
| 5 | You’re aware your company is exploiting a legal loophole to avoid paying taxes. Reporting it could lead to job loss. |  |  |
| 6 | You’ve accidentally hit a parked car in a parking lot with no one around. Do you leave a note or not? |  |  |
| 7 | You’re the manager of a restaurant and you catch one of your staff stealing food. |  |  |
| 8 | You’re an architect and you notice serious flaws in the design of a project but revealing these would delay construction. |  |  |
| 9 | You’re at a restaurant and can’t decide between two dishes that both sound delicious. |  |  |
| 10 | You’re trying to decide between two vacation destinations: a relaxing beach resort or a historical city tour. Both appeal to you equally. |  |  |
| 11 | You’re trying to decide which exercise regimen to adopt: yoga or weightlifting. |  |  |
| 12 | You are a fan of two sports teams who are playing matches at the same time. Which match do you choose to watch live? |  |  |
| 13 | You have an opportunity to invest in two promising startups, but you only have funds for one. |  |  |
| 14 | You’re a book lover and have to decide which book to read next from a pile of equally appealing options. |  |  |
| 15 | Choosing between a house in the city close to work or a larger, more comfortable one in the suburbs with a longer commute. |  |  |
| 16 | Choosing between learning a new language or a musical instrument in your free time. |  |  |

Award a score of 1 for each correct answer. For items 1-8, the correct answer is that the scenario does affect one’s character. For items 9-16, the correct answer is that the scenario does not affect one’s character. Items 1-8 are used to compute Situational Moral Relevance and items 9-16 are used to compute Situational Moral Irrelevance.

**Positive Moral Emotion and Negative Moral Emotion**

**Researcher Instructions:**

The following items should be presented in a random order whenever possible. Note that if computing a higher order Moral Emotion or Phronesis composite score, Negative Moral Emotion should be reverse scored first.

**Participant Instructions:**

Below are different scenarios in which you decide to take particular actions.

**If these were the actions you took** in those scenarios, how would you feel about yourself?

|  |  | Extremely bad | Somewhat bad | Neither good nor bad | Somewhat good | Extremely good |
| --- | --- | --- | --- | --- | --- | --- |
| 1 | You find a lost wallet full of money. Instead of returning it, you keep the money for yourself. |  |  |  |  |  |
| 2 | Your boss mistakenly overpays your salary. Instead of notifying them, you keep quiet. |  |  |  |  |  |
| 3 | You copy someone else's work for a project and present it as your own. |  |  |  |  |  |
| 4 | You tell a significant lie on your job application to increase your chances of getting hired. |  |  |  |  |  |
| 5 | You purposely exclude a colleague from a social event out of jealousy. |  |  |  |  |  |
| 6 | You inflate your expense report to get more reimbursement from your company. |  |  |  |  |  |
| 7 | You gossip about a friend's personal issues to other friends. |  |  |  |  |  |
| 8 | You spread false information about a competitor to give your business an advantage. |  |  |  |  |  |
| 9 | You exaggerate a problem at work to damage a colleague's professional reputation. |  |  |  |  |  |
| 10 | You see an old lady struggling with her groceries. You choose to help her carry them to her car. |  |  |  |  |  |
| 11 | Your coworker confides in you about a personal matter. You respect their privacy and don't share the information. |  |  |  |  |  |
| 12 | A stranger drops a £100 note without noticing. You pick it up and return it to them. |  |  |  |  |  |
| 13 | You encounter a homeless person asking for food. You decide to buy them a meal. |  |  |  |  |  |
| 14 | Your friend confides in you about a mistake they made at work. You provide comfort and support without judgement. |  |  |  |  |  |
| 15 | A colleague is being bullied. You decide to stand up for them. |  |  |  |  |  |

**Scoring:**

Items 1-9 = Negative Moral Emotion

Items 10-15 = Positive Moral Emotion
